# Process Feasibility Analysis of Waste Biomass Valorization to Biochar and Bio-Oil via Slow and Fast Pyrolysis

**DOI:** 10.1021/acs.energyfuels.5c05655

**Published:** 2025-12-25

**Authors:** Geetanjali Yadav, Patrick Lamers

**Affiliations:** † Catalytic Carbon Transformation and Scale-Up Center, 53405 National Renewable Energy Laboratory, Golden, Colorado 80401, United States; ‡ Strategic Energy Analysis Center, 53405 National Renewable Energy Laboratory, Golden, Colorado 80401, United States

## Abstract

The United States has abundant biomass and waste feedstock to support the nation’s energy addition and affordability targets. Pyrolysis, a thermochemical conversion process, decomposes lignocellulosic feedstocks into liquid, solid, and gaseous fuels that can contribute to the domestic production of biofuels, biopower, and bioproducts. Growing private sector interest in this technology is a key motivation for this comprehensive techno-economic process modeling analysis of a respective biorefinery that includes feedstock preprocessing, slow and fast pyrolysis, and product separation to bio-oil, biochar, and syngas hydrocarbons. Results show that biochar from slow pyrolysis could achieve minimum selling prices (MSPs) of $188–$260/t, competitive with reported market values, while bio-oil from fast pyrolysis is estimated to yield MSPs of $6.49–$9.68/GGE, approximately twice conventional fuel benchmarks. Sensitivity analysis identifies feedstock cost, product yield, and scale as primary cost drivers, while scenarios involving biochar carbon credits and high value applications may substantially improve economics. Overall, these results suggest that continued innovation in feedstock logistics, process integration, and market development will be critical to achieving economically viable and scalable bioproducts.

## Graphical abstract

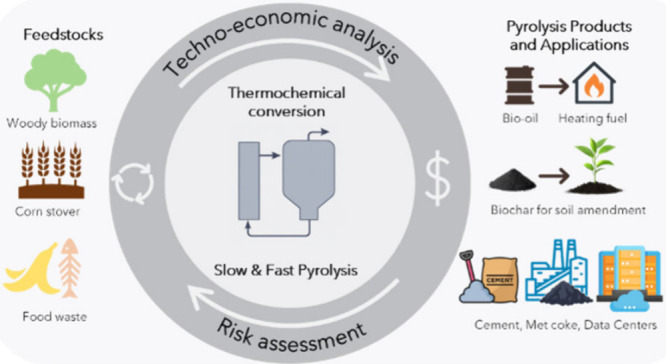

## Introduction

The United States (U.S.) has an abundant biomass and waste feedstock potential which can support the nation’s quest to energy addition and affordability via the domestic production of biofuels, biopower, and bioproducts.^1,2^ In 2023, biomass accounted for about 5% of U.S. energy consumption (4,978 TBtu).^3^ Despite growing interest in biomass conversion technologies, a process-level understanding and comprehensive modeling remain to evaluate technical performance, guide design, and inform scale-up for private sector deployment.

Agricultural and forestry residues represent the largest share of this resource base.^4–6^ In 2022, 127 million metric tons (MMT) of agricultural residues and 28 MMT of forest residues were not utilized for energy or material use.^1^ Instead, these resources ended up in landfills, compost, or fodder, or were burned in fields, contributing to pollution and the loss of valuable carbon. While the carbon and energy contents of those materials alone cannot meet the full energy needs of the transportation or power sectors, their recovery could still deliver meaningful environmental and economic benefits. Conventional practices such as lignin combustion for heat and power can miss opportunities for higher-value uses, including chemical and liquid fuel synthesis or carbon storage,^7^ highlighting the need for strategies that maximize biomass utilization and enhance carbon retention through improved process design and system-level integration.

Pyrolysis is a thermochemical conversion process in which lignocellulosic feedstocks are decomposed under oxygen-limited conditions, typically within a temperature range of 300–700 °C, to produce liquid (bio-oil), solid (biochar), and gaseous (noncondensable gases, NCGs) products.^8,9^ The process is broadly categorized into slow or fast pyrolysis, depending on temperature,^10,11^ heating rate,^12^ residence time,^13^ and reactor configuration.^14^ Slow pyrolysis operates at low heating rates (0.1–1 °C/s) and moderate temperatures (300–500 °C) with vapor residence times ranging from minutes to hours, favoring biochar production.^8^ In contrast, fast pyrolysis involves high heating rates (10–1000 °C/s) at 400–700 °C, with vapor residence times typically below 2 s, maximizing bio-oil yield.^7^ The product distribution strongly depends on the pyrolysis mode. Fast pyrolysis generally yields 50–75 wt % bio-oil, 15–25 wt % biochar, and 10–20 wt % NCGs,^14^ whereas slow pyrolysis produces 10–25 wt % bio-oil, 30–65 wt % biochar, and 5–15 wt % NCGs.^8,15,16^ These ranges reflect typical values reported across diverse feedstocks and operating conditions, and actual yields may vary depending on the temperature, heating rate, and reactor configuration.

Raw pyrolysis oil has limited direct market value due to the presence of oxygenated hydrocarbons and a significant amount of water, typically ranging from 15 to 30%, originating from both the initial feedstock moisture and water produced during pyrolysis reactions.^17^ However, it can be upgraded into transportation fuels such as diesel, gasoline, marine fuel, or jet fuel,^18^ or alternatively sequestered underground for long-term carbon storage.^19^

Biochar is widely utilized as a soil amendment, improving soil fertility, nutrient retention, and crop productivity.^20^ Depending on feedstock, processing conditions, and plant scale, biochar market prices range from $91 to $350 per ton for large-scale facilities, whereas it is reported to range from $200 to $1000 per ton for most of the small to moderate scale producers in North America.^21–24^ Beyond agronomic uses, biochar has recently gained strong interest in voluntary carbon markets (VCMs) as a carbon dioxide removal (CDR) pathway, generating additional revenues through the sale of carbon credits (CC).^24,25^ These credits are typically purchased by corporations to offer personal emission offsets to consumers (e.g., airlines) or to meet voluntary corporate emission reduction targets by offsetting hard-to-abate emissions.^26^ Biochar is among the few CDR solutions currently available at scale, achieving annual carbon removals of approximately 0.65 MMT of CO_2_, with credit values ranging from $42 to $250/MT in 2024 (average ∼$131/MT).^26,27^ Additionally, it can serve as a renewable energy source to support the growing energy demand of data centers, either directly as energy or as a sustainable material for partial cement replacement.^28^ It can also substitute metallurgical coke in iron and steelmaking, addressing emissions in hard-to-abate sectors.^29,30^ The pyrolysis gases or NCGs can be combusted to produce electricity and process heat, which may either be exported to the grid or used to satisfy the on-site energy requirements, thereby improving plant energy self-sufficiency.

The demonstrated multifunctional value of pyrolysis products and their synergies with carbon removal have heightened private sector interest in the cost and performance of biomass pyrolysis. While prior analyses have examined process operations including the impact of coproducts, plant size, feedstock, and operating conditions, a comprehensive and transparent techno-economic analysis (TEA) of biomass pyrolysis for fuels and carbon-negative products integrated with CDR strategies is still lacking.^7,8,31,32^ To that end, we developed a process model of a biomass biorefinery incorporating feedstock preprocessing, slow and fast pyrolysis, and product separation to bio-oil, biochar, and syngas hydrocarbons, using three representative feedstocks: woody biomass, agricultural residues, and organic waste. Leveraging extensive experimental data available in the literature,^33–36^ we designed a conceptually feasible biorefinery configuration with a 30-year operational horizon, incorporating realistic process efficiencies and capital scaling factors. The fast pyrolysis case was examined in greater detail, highlighting the potential of coproduct biochar for additional value-added applications, such as partial substitution of cement additives and metallurgical coke. Furthermore, policy incentives that could provide additional economic benefits to qualifying product streams were explored. Sensitivity and scenario analyses were conducted to identify key costs and performance drivers.

## Methods: Process and Economic Model Construction

We modeled two distinct base case pyrolysis systems using Aspen Plus v14 software: a slow pyrolysis (SP) configuration designed to maximize biochar production for soil amendment applications and a fast pyrolysis (FP) system optimized for bio-oil production. A process flow diagram for the two pyrolysis cases is presented in Figure 1. Three representative feedstocks were considered: (1) herbaceous biomass, represented by agricultural residues such as corn stover (CS); (2) forest woody biomass, primarily pine wood (PW); and (3) organic waste, represented by food waste (FW), which constitutes the second-largest waste stream in the U.S by volume.^37^ The proximate and ultimate analyses of these feedstocks, along with the key pyrolysis process parameters adopted from the literature,^11,35,38–41^ are summarized in Table 1.

**Table 1:** Proximate and Ultimate Analysis of the Feedstock and Pyrolysis Process Conditions Considered in the Study^a^

parameters	units	slow pyrolysis			fast pyrolysis		
feedstock type		woody biomass	herbaceous biomass	organic solid waste	woody biomass	herbaceous biomass	organic solid waste
feedstock		pine wood	corn stover	food waste	pine wood	corn stover	food waste
Ultimate Analysis of Biomass Feedstock							
C	wt %	47.51	48.8	44.69	50.30	48.99	51.31
H	wt %	6.52	6.41	4.49	6.30	5.25	6.25
O	wt %	45.87	44.1	46.33	43.30	44.70	37.49
N	wt %	0.095	0.65	4.27	0.10	0.83	4.56
S	wt %	0.005	0.04	0.22	0.00	0.23	0.39
O/C	molar ratio	0.724	0.678	0.778	0.646	0.684	0.548
H/C	molar ratio	1.647	1.576	1.206	1.503	1.286	1.462
Proximate Analysis of Biomass Feedstock							
volatile matter	dry basis	81.12	75.37	84.56	80.30	79.70	86.48
ash content	dry basis	0.86	5.43	9.86	0.86	5.50	8.81
fixed carbon	dry basis	18.02	19.20	5.59	18.84	14.80	4.71
heating value of feedstock (HHV)	MJ/kg	18.59	16.59	17.00	18.07	17.10	16.56
Pyrolysis Conditions							
temp, *T*_pyr_	°C	300	300	300	500	500	600
pyrolysis vapor residence time	h, min or second	2 h	29 min	30 min	2 s	1.5 s	6 s
pressure, *P*_pyr_	bar	1					
plant size	dry MTPD	2000					
ref		Williams and Besler^38^	Soka and Oyekola^39^	Patra et al.^40^	DeSisto et al.^41^	Shah et al.^45^	Qing et al.^11^

a Although the feedstocks represent three distinct biomass classes, the data were compiled from six different literature sources. Consequently, variations in proximate and ultimate compositions were observed, and minor adjustments (<3%) were made to proximate composition values (O, H, fixed carbon) as well as product yields to ensure 100% mass closure and consistency across data sources; elemental C and N values were unchanged.

**Figure 1:**
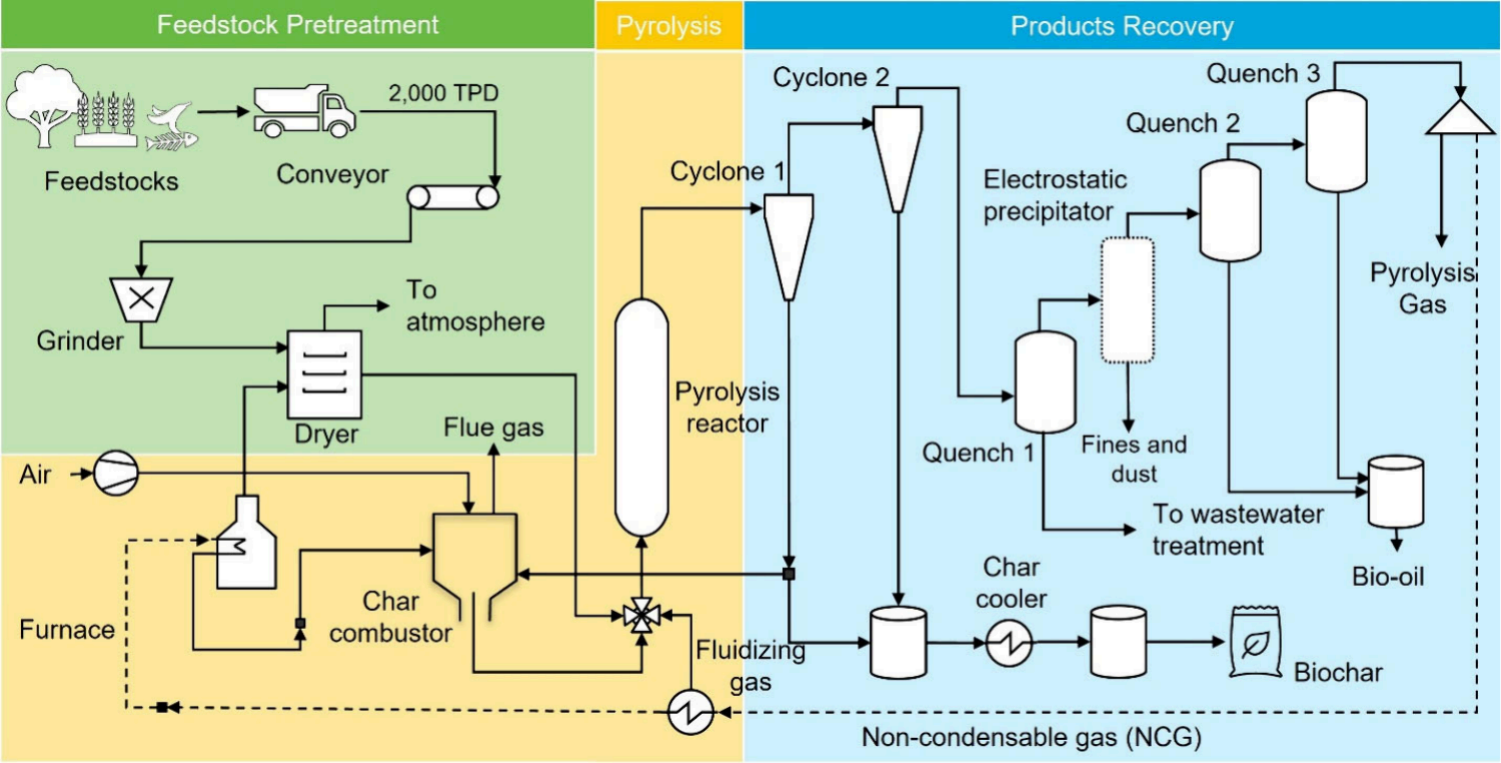
Simplified process flow diagram of the modeled biomass pyrolysis systems. Feedstock pretreatment: Biomass undergoes size reduction and drying. Pyrolysis: The pretreated biomass is subjected to thermochemical conversion to produce bio-oil, biochar, and NCGs. Products recovery: The reactor effluent is cooled, and solids are separated from the gaseous stream by using cyclones. The gaseous stream is subsequently quenched to recover bio-oil, while remaining NCGs are primarily utilized for on-site energy. An electrostatic precipitator (ESP) can optionally be installed downstream of the cyclones to capture fine char particles from the pyrolysis vapors, improving bio-oil quality.

Based on the availability of the feedstocks in the U.S., woody biomass and corn stover are assumed to be delivered to the pyrolysis facility by truck at $81/ton and $87/ton,^42^ respectively, while food waste is available at $50/ton.^43,44^ These costs include expenses associated with collection, preprocessing, and storage prior to pyrolysis, including both capital and operating expenditures related to biomass handling at approximately 30 wt % moisture (as received) and size reduction to 2–6 mm. The pyrolysis facility is designed to process 2000 dry metric tons per day (tpd) of feedstock. This size was selected to enable a direct techno-economic comparison between slow and fast pyrolysis pathways under equivalent feedstock throughput, while capturing key differences in product yields and coproduct economics. Within the facility, biomass is fed from a hopper and passes through a cross-flow dryer, reducing its moisture content to approximately 10 wt % before being introduced via a screw feeder into the pyrolysis reactor for thermochemical conversion.

After pretreatment, the feedstock pellets are introduced into the pyrolysis reactor. A fixed-bed reactor is employed for SP,^46^ while a fluidized-bed reactor is used for fast pyrolysis.^47^ The SP reactor operates at 300 °C (also termed torrefaction at this temperature), whereas the FP reactor operates at 500–600 °C, both under atmospheric pressure. Feedstock composition, product yields, and process parameters were derived from published literature and adjusted, where necessary, to ensure complete mass and energy balance closure.^11,35,38–41^ In the FP system, NCGs are recycled to provide the necessary fluidization in the pyrolysis reactor.^48^ Additionally, a portion of the NCGs and bio-oil is utilized to supply heat to the reactor when it is required.

Following pyrolysis, the reactor effluents pass through two cyclones in series to remove (>99.9%) solid char and fine particulates from the hot vapor stream.^49^ The vapors are then condensed to recover liquid bio-oil, while the remaining NCGs are either recycled or combusted to generate heat, which can be further converted to electricity via a conventional steam turbine. The revenue contribution from coproducts is accounted for in determining the MSP of the primary product.

Material and energy balances derived from the process models were used to estimate the equipment sizing and the corresponding capital investment for the pyrolysis configurations. Data on raw materials, utilities, labor, and maintenance cost, and other factors informed the calculation of annual operating expenses. A discounted cash flow rate-of-return (DCFROR) analysis (financial parameters in Table S1) was applied to determine the MSP of the principal product on a 2020 United States dollar cost basis, ensuring a 10% internal rate of return (IRR) over a 30-year plant lifetime and a net present value (NPV) of zero. Coproducts such as bio-oil and biochar were accounted for as credits in the TEA. In the techno-economic analysis, the coproduct is defined relative to the principal product: when biochar is the principal product (slow pyrolysis), bio-oil and NCGs are treated as coproducts, and conversely, when bio-oil is the principal product (fast pyrolysis), biochar and NCGs are treated as the coproduct. Bio-oil was priced comparably to No. 2 heating oil, using a 5-year average market value of $3.91/gal (2018–2022),^50^ while the biochar price was conservatively set at $100 per metric ton (t), reflecting typical values for soil amendment applications.

## Results

### Slow Pyrolysis

The total capital investment (TCI) for a 2000 dry metric tpd SP facility is estimated at $128MM (Figure 2A) for PW feedstock. The pyrolysis section contributes the largest share of the capital cost (74%), driven primarily by the reactor system, followed by outside battery limits (OSBL) investment and product recovery. The OSBL accounts for 25% of the inside battery limits (ISBL), representing the additional infrastructure required to integrate the over-the-fence utilities and supporting systems.^51^ The annual operating expense (OPEX) for the SP plant is $85MM, which decreases to $61MM after accounting for coproduct credits (Figure 2B).

**Figure 2:**
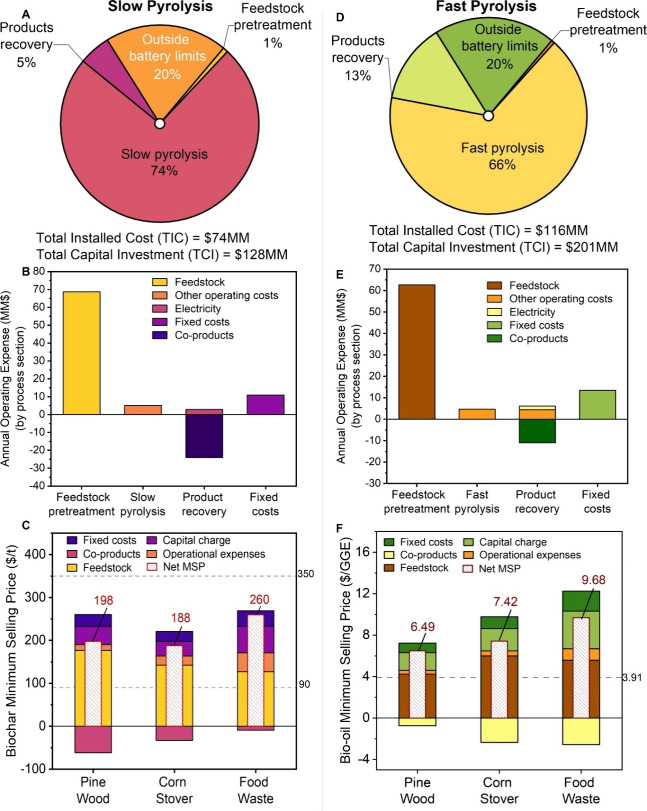
Techno-economic results for slow and fast pyrolysis processes at 2000 dry metric tpd scale. (A, D) Capital cost breakdown by process section, showing total installed capital of $74MM and $116MM and TCI of $128MM and $201MM for SP and FP, respectively, for PW feedstock. (B, E) Annual operating expenses for slow and fast pyrolysis, disaggregated by process section (Net = $61MM and $70MM per year, respectively), with the largest contribution from feedstock costs; fixed costs include labor and overhead for PW. Note: The positive *y*-axis values for the product recovery category are scaled by a factor of 10 for the sake of clarity. (C, F) MSP breakdown for biochar (C), with the conventional market price indicated by a range flanked by dotted lines, and for bio-oil (F), with the conventional price shown as a dotted line at $3.91/GGE (assuming energy equivalence with gasoline). CAPEX and OPEX for CS and FW in SP and that in FP are shown in Figure S2–S5. All data shown in this figure, including the breakdown of capital, operational expenses, and MSP with fixed and variable costs, are included in Tables S3–S17.

The MSP of biochar in the base case is $198/t, $188/t, and $260/t for PW, CS, and FW feedstocks, respectively (Figure 2C). These values are tied primarily to the product yields from the feedstocks (Figure S1) and fall within the reported market range of $91–$350/t.^23^ Among the cost contributors, feedstock cost represents the primary economic driver, followed by capital investment and fixed operating costs. The capital expenditures (CAPEX) and OPEX for CS and FW feedstocks are provided in Figures S2 and S3. Coproducts were accounted for as credits based on their lower heating values and 5-year average market price.^52^

### Fast Pyrolysis

The FP system demonstrates a higher overall capital intensity, with a TCI of $201MM (Figure 2D) for PW feedstock. Similar to SP, the pyrolysis section is the largest contributor (∼66%) to capital cost, primarily due to the high cost of the reactor and the fluidized gas compressor. The total annual OPEX for the FP process is $81.4MM before coproduct credits (Figure 2E), and it reduces to $70MM after coproduct credits, which is higher than that of the SP process ($61MM) due to the lower value coproduct, i.e., biochar. The MSP of bio-oil is estimated at $6.49, $7.42, and $9.68 per gasoline-gallon equivalent (GGE) for PW, CS, and FW feedstocks, respectively, and breakdown is shown in Figure 2F. These values are 1.7–2.5 times higher than the five-year average retail price of No. 2 fuel oil ($3.91/GGE),^53^ reflecting the lower energy density and compositional complexity of pyrolytic bio-oils, which contain 25–35 wt % water and a high concentration of oxygenated compounds.^54^ As a result, their heating value is typically 30–50% that of conventional fossil fuels, necessitating costly upgrading steps.^54,55^ Table S2 compares the quality of bioproducts from each pyrolysis mode and its impact on the quality of these products. Feedstock cost remains the factor contributing the most to the MSP, followed by capital and fixed operating costs. The CAPEX and OPEX for CS and FW feedstocks are provided in Figures S4 and S5.

#### Sensitivity Analysis

A univariate sensitivity analysis was conducted to identify the key parameters that influence the economic viability of biomass pyrolysis, as shown in the tornado plot (Figure 3). In the base case of SP with three different feedstocks, the MSP of the biochar product was found to be most sensitive to feedstock cost (Figure 3A–C). Increasing the cost of woody and agricultural residues from $81/t and $87/t (base case) to $122/t increased the MSP by 30–45%, whereas for organic waste, raising the cost to $70/t increased the MSP by 20%. Note that tipping fees were not considered for waste handling. This strong influence of feedstock price on pyrolysis economics aligns with previous TEA studies, which consistently identify biomass cost as the dominant driver in thermochemical biomass conversion systems.^31,39,51,56^ More details on the feedstock prices and the rationale for choosing the low and high range of parameters in the sensitivity analysis are provided in Table S18.

**Figure 3:**
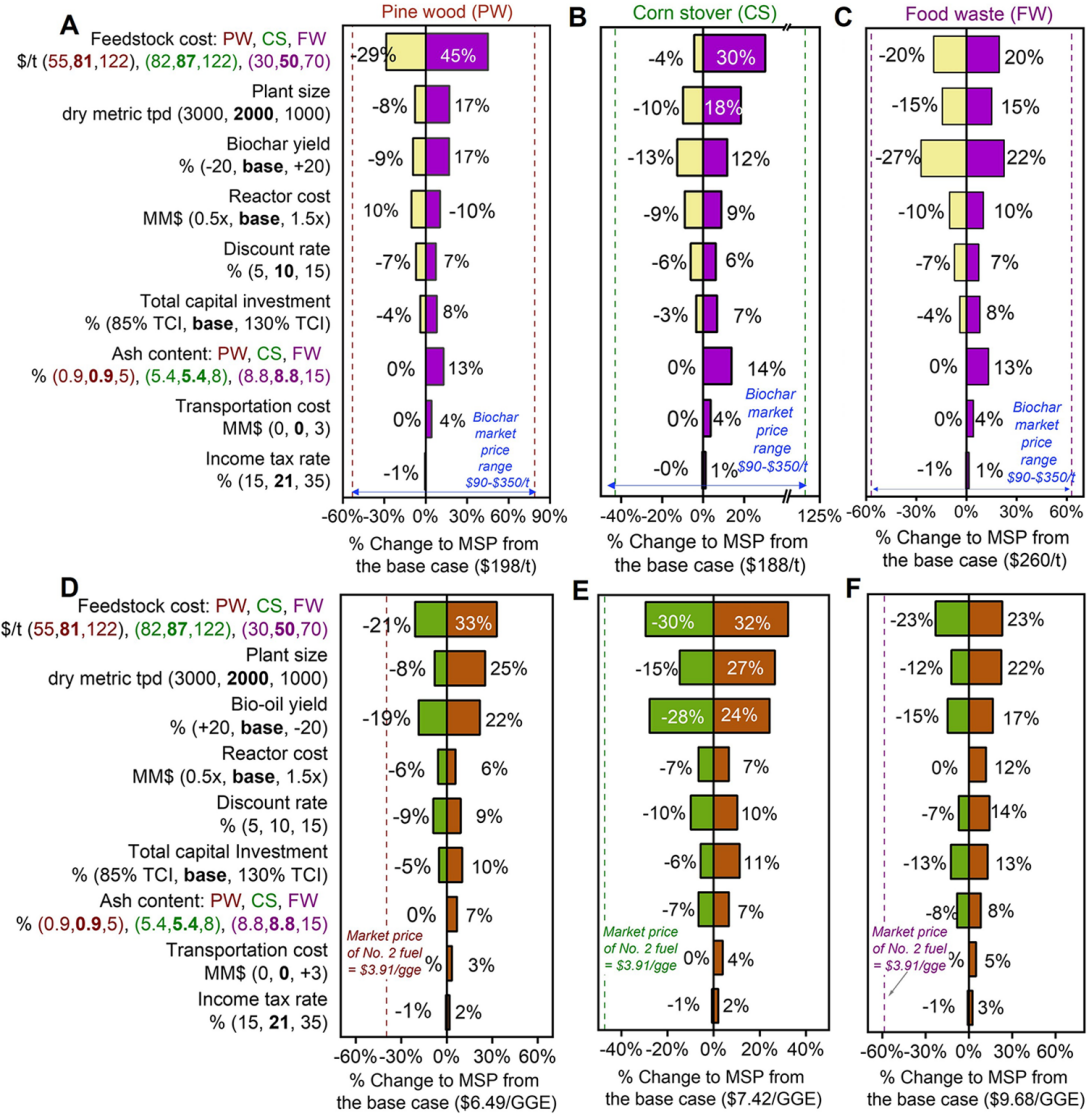
TEA sensitivity analysis results. Single-point TEA sensitivity analyses showing the effect of key process and financial parameters on the MSP of biochar (A–C, SP) and bio-oil (D–F, FP) for wood (pine wood, PW), herbaceous (corn stover, CS), and organic (food waste, FW) feedstocks. Parameter values shown in the leftmost panels (A and D) are representative of each feedstock category, with specific values explicitly indicated where they differ among feedstocks. The central line denotes the baseline MSP. The dotted lines flanking the baseline in panels A–C indicate the market price range of biochar, while those in panels D–F mark the market price of No. 2 fuel oil. Rationale for selecting the sensitivity range values is provided in Table S19. All data shown in this figure is included in Tables S20–S25.

Product yield emerged as another critical cost driver. In the base case SP scenarios, biochar yields were 54%, 67%, and 52% for PW, CS, and FW, respectively^38–40^ (Figure S1A). A 20% increase in biochar yield decreases the MSP by up to 22%, while a 20% decrease in biochar yield increases it by as much as 27% across the cases. This inverse relationship contrasts with that observed for bio-oil under FP conditions, where higher bio-oil yields reduce the MSP by 15–28% (Figure 3D–F). This difference stems from the intrinsic carbon trade-off between solid and liquid phases and products during thermochemical conversion. Greater carbon retention in biochar can constrain overall economic returns unless the biochar is valorized through high-value applications, carbon sequestration credits, or policy-driven incentives.^57,58^ Conversely, in FP, higher bio-oil yields directly enhance process revenue, improving economic performance through greater product throughput and reduced fixed and variable costs per unit product. This trade-off aligns with studies showing that fast pyrolysis, which favors bio-oil, often yields higher economic returns than biochar-focused slow pyrolysis.^56,59^ Product yield distributions across all process scenarios are presented in Figure S1.

Plant scale also exerted a strong influence on the MSP. Assuming no process modifications and a constant feedstock price, increasing the plant capacity from 2000 to 3000 dry metric tpd reduced the MSP by 8–15% due to economies of scale.^60^ This reduction results from fixed costs such as equipment, labor, and overhead being distributed across greater product output, thereby decreasing the unit production cost.^47,51,60^ Ash content was included as an additional sensitivity parameter in this analysis to capture its effect on process economics. Higher ash levels, especially in waste feedstocks, reduce carbon conversion and product yields, increasing MSP by 7–14% across both slow and fast pyrolysis routes, consistent with the observed rise in MSP as ash content increases from 1% to 7%.^61^

Feedstock-related costs, particularly those associated with logistics and preprocessing, were also evaluated. Although transportation costs were not included in the base case, a sensitivity scenario was modeled for long-distance feedstock transport (500 miles) at $0.25/ton-mile.^62^ This is particularly relevant for a large-scale facility sourcing feedstock over extended distances. This scenario could increase the MSP by up to 5% in SP and 8% in FP cases, underscoring the importance of localized feedstock sourcing to maintain the cost competitiveness. Additional sensitivity parameters, including discount rate, reactor cost, additional feed preprocessing, and pyrolysis reactor pressure exhibited marginal effects on the principal product’s MSP.

#### Effect of Emerging Voluntary Corporate Carbon Markets

While there is a recognized potential for biochar as a soil amendment, market take-up has been slower than anticipated. However, biochar is gaining increasing prominence in the voluntary carbon market as the production process has a higher technology-readiness level compared to other CDR options and could be viable for at-scale deployment.^63^ Corporate interests in biochar are driven by the relatively lower CC price and biochar’s co-benefits for soil health in agricultural or other high-value applications such as cement adhesive,^64,65^ as a substitute for metallurgical coke,^30,66^ and in wastewater treatment.^67^

Biochar’s market value as a CC agent is directly linked to its long-term stability, which is primarily determined by its oxygen-to-carbon (O/C) and hydrogen-to-carbon (H/C) molar ratios.^33,68,69^ Biochars with O/C ≤ 0.2 are considered highly stable or recalcitrant in topsoil, with estimated half-lives >1000 years.^68^ Those with O/C between 0.2 and 0.6 are moderately stable (half-lives: 100–1000 years), while O/C > 0.6 indicates lower stability (<100 years) and is usually an indication of non-pyrolytic chars or pyrolysis deficiencies. The H/C ratio is considered a more robust indicator of durability, as it remains relatively unaffected even during oxidative conditions.^68,69^ An H/C < 0.7 signifies greater aromaticity and structural integrity,^70^ indicating higher stability and persistence suitable for long-term carbon sequestration, whereas H/C > 0.7 suggests reduced persistence in the environment due to the presence of more labile carbon forms. These ratios are also part of biochar quality criteria defined by international standards. The International Biochar Initiative (IBI) and the European Biochar Certificate (EBC) specify maximum O/C ratios (typically ≤ 0.4) and H/C ratios <0.7 for agronomic use.^71^ In addition to the O/C and H/C ratios, these regulatory standards specify additional biochar properties including volatile organic compounds, nutrient content (N, P, K, Mg, Ca, Fe), heavy metals, pH, bulk density, electrical conductivity, water-holding capacity, surface area, porosity, and polycyclic aromatic hydrocarbons (PAHs). These properties are critical for assessing biochar performance, compliance with regulatory criteria, and potential environmental impacts.^71^

Figure 4A maps the distribution of the O/C and H/C molar ratios for a diverse set of biomass and biomass-derived biochar evaluated in this study. The plot delineates distinct stability regimes such as high, moderate, and low based on established thresholds, providing a practical framework for assessing and optimizing biochar durability. In comparison to the O/C and H/C ratios of the original feedstocks, these values were lower for the biochar produced from those feedstocks, indicating the loss of H and O and C enrichment. This visualization (Figure 4A) aids in guiding pyrolysis process parameters and feedstock selection to engineer biochar with tailored stability profiles suitable for specific environmental or agronomic applications.

**Figure 4:**
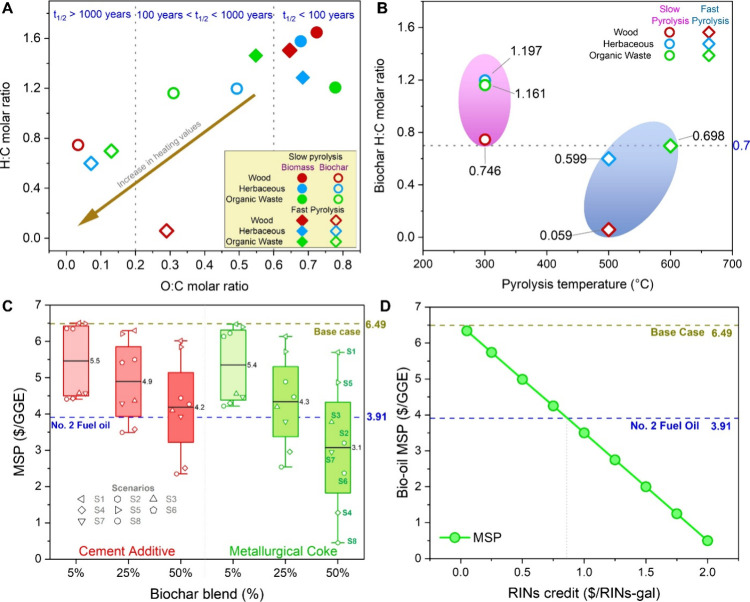
(A) Van Krevelen diagram showing the variation in H/C and O/C molar ratios of biomass feedstocks and resultant biochars from slow and fast pyrolysis. The arrow indicates increasing aromaticity and carbon stability with decreasing O:C and H:C ratios, corresponding to longer half-life ranges based on Spokas et al.^68^ (B) Dependence of biochar H:C ratio on pyrolysis temperature. The horizontal dotted line denotes H/C = 0.7, indicating stable biochar. (C) Bio-oil MSP under varying biochar blending ratios and utilization routes: cement additive (red), and metallurgical coke (green). Each box plot represents eight scenarios (S1–S8) represented by different symbols. The box plot displays the distribution of the MSPs, with the lower and upper edges of each box representing the Q1 (25%) and Q3 (75%) quartiles, respectively. The line inside the box indicates the mean value. The interquartile range (IQR), defined as Q3–Q1, captures the middle 50% of the data, while the whiskers extend to the minimum and maximum values of MSPs. Dashed lines denote the base case ($6.49/GGE) and No. 2 fuel oil benchmark ($3.91/GGE). Detailed methodology is provided in the Supporting Information, and the parameters are shown in Tables S26–S29. (D) Reduction of bio-oil MSP with increasing renewable identification number (RINs) credit ($/RINs-gal), approaching fuel oil parity atapproximately $2/RINs-gal.

Figure 4B illustrates the influence of the temperature on the biochar stability. Biochar located within the blue zone exhibits lower H/C molar ratios compared to those in the pink zone, indicating greater aromaticity and structural stability.^69^ These trends correspond to biochar produced via FP, a high temperature process that rapidly releases volatile matter, promotes pore formation, enhances aromatic cluster development, and increases overall recalcitrance factors that contribute to improved long-term stability in soil environments.^72^

Next, Figure 4C,D present the techno-economic implications of coproduct valorization and policy incentive mechanisms on the MSP of bio-oil. No carbon credits were included in the base case. To evaluate the economic potential of biochar as a coproduct in industrial applications, Figure 4C presents box plots examining biochar valorization under two utilization pathways, cement additive and metallurgical coke substitution, across eight market scenarios (S1–S8). These scenarios vary biochar price ($90–$350/t), end-use product price (cement = $100–160/t; coke = $130–430/t), and carbon credit (CC = $50–350/t). For reference, U.S. cement and metallurgical coke prices range from $100–160/t,^73^ and $130–430/t,^74^ respectively, with associated emission intensities of 0.9 ^75^ and 3.0 tCO_2_ per ton.^76^ These sectors are among the largest industrial sources of CO_2_, making reductions in emissions critical for meeting global climate targets. Each box plot represents eight combinations of these variables, capturing both the mean and variability in the MSP outcomes at 5%, 25%, and 50% biochar blending levels. The increasing whisker spread with blending ratio indicates that greater biochar substitution enhances cost-reduction potential but also increases exposure to market fluctuations. This analysis assumes increased biochar substitution provides similar proportional benefits; actual effects may vary with dosage and curing conditions. Biochar with high fixed carbon (>80 wt %), low moisture (1–5 wt %), high calorific value (30–32 MJ kg^–1^) meets the performance requirements reported for metallurgical coke, whereas high water absorption property and high porosity/surface area (≈58% and 300–450 m^2^ g^–1^), microfiller effect, and beneficial oxides (SiO_2_, Al_2_O_3_, CaO, K_2_O) improve cement hydration and the formation of binding gels, resulting in higher compressive, tensile, and flexural strength at low doses.^77^ These performance improvements are particularly relevant for infrastructure supporting high-energy facilities such as data centers, where concrete with biochar can reduce embodied CO_2_ while maintaining structural performance.^28^ The detailed methodology is provided in the Supporting Information, and the parameters are shown in Tables S26–S29.

In the cement additive pathway (red), the MSP of bio-oil decreases progressively with an increasing biochar blending ratio and favorable market conditions. Relative to the base case ($6.49/GGE), MSP reductions of 32% to 64% were achieved at 5–50% blending levels, primarily due to the displacement of clinkers and associated CO_2_ emissions (Figure 4C). Specifically, S1 and S2 correspond to low biochar prices and low cement prices, with low and high CC, respectively, while S3 and S4 represent high biochar prices under the same low cement price, again with low and high CC. All four scenarios share a lower fixed cement price than scenarios S5–S8, which are evaluated at a higher cement price ($160/t). The largest reduction occurs in S8 (high biochar price, high cement price, high CC), where the MSP decreases by 64% to $2.35/GGE at 50% biochar blending, reflecting the combined benefit of high coproduct revenue and strong carbon credits. Conversely, low-value markets (e.g., S1) yield minimal improvement (<1%) relative to the base case, indicating that economic gains are highly sensitive to cement price and CC. It is important to note that most cement scenarios, even at 50% blending, still hover near or slightly above the No. 2 fuel-oil benchmark (3.91 $/GGE), except under the most optimiztic conditions (S4 and S8).

Further, the metallurgical coke substitution in the iron and steel industry pathway (green) demonstrates a stronger reduction in MSP due to the higher market value and carbon intensity of metallurgical coke. MSP reductions range from 4% to 93% across scenarios with the lowest value of $0.45/GGE achieved in S8 at 50% blending. Even at moderate blending (25%), average MSPs ($4.30/GGE) reduce by up to 33% compared to the base case, indicating strong economic leverage through this valorization route. The majority of 50% coke-substitution cases fall below the No. 2 fuel-oil parity line, demonstrating that even with greater uncertainty, this pathway consistently offers superior economic potential compared to the cement route.

Figure 4D evaluates the impact of policy incentives such as Renewable Identification Number (RIN) credits under the U.S. Renewable Fuel Standard 2 (RFS2) program,^78^ on the MSP of bio-oil. The analysis assumes that the base case does not include any RIN credit allocation. Incremental RINs values, ranging from $0.05 to $2.0/RINs-gal, are applied to quantify their influence on bio-oil market competitiveness. The MSP shows a nearly linear decline with increasing RIN value, decreasing from the base case of $6.49/GGE to $3.49/GGE at a RIN credit of $1.0/RINs-gal, and further approaching parity with No. 2 fuel oil ($3.91/GGE) at approximately $2.0/RINs-gal.

This trend underscores the sensitivity of bio-oil economics to renewable fuel policy mechanisms. Under realistic credit conditions for D3/D7 cellulosic biofuels, where D3 refers to cellulosic ethanol and D7 refers to cellulosic diesel, jet, or other nonethanol fuels, RINs can offset production costs substantially, enabling cost-competitive bio-oilproduction without requiring extreme feedstock or coproduct assumptions. The results indicate that the integration of policy instruments such as RINs, CC, or low-carbon fuel incentives can directly enhance the profitability of pyrolysis-derived fuels, bridging the economic gap between advanced biofuels and conventional fossil-derived fuels.

Lastly, a multivariate analysis (Figure 5A–C) was conducted to evaluate how CO_2_ or CC price ($50–350/tCO_2_)^79^ and recalcitrant carbon fraction (RCF) of biochar may affect the MSP of bio-oil from fast pyrolysis of woody biomass.^68^ The RCF is tied to the biochar’s O/C atomic ratio, where O:C < 0.2 corresponds to a half-life exceeding 1000 years (RCF > 80%), while O:C > 0.6 yields RCF < 50%. These parameters were varied to assess the economic value of biochar as a carbon sequestration agent. Measurement, Monitoring, Reporting, and Verification (MMRV) costs were included to realistically account for expenses associated with validating biochar carbon credits, ensuring that estimated CC revenues reflect true market conditions. MMRV costs ranged from $12–40/tCO_2_, and biochar transportation costs from $5–40/tCO_2_, consistent with reported in the literature, $20/t for direct air capture and $40/t for enhanced rock weathering.^80,81^ A detailed methodology is provided in the Supporting Information.

**Figure 5:**
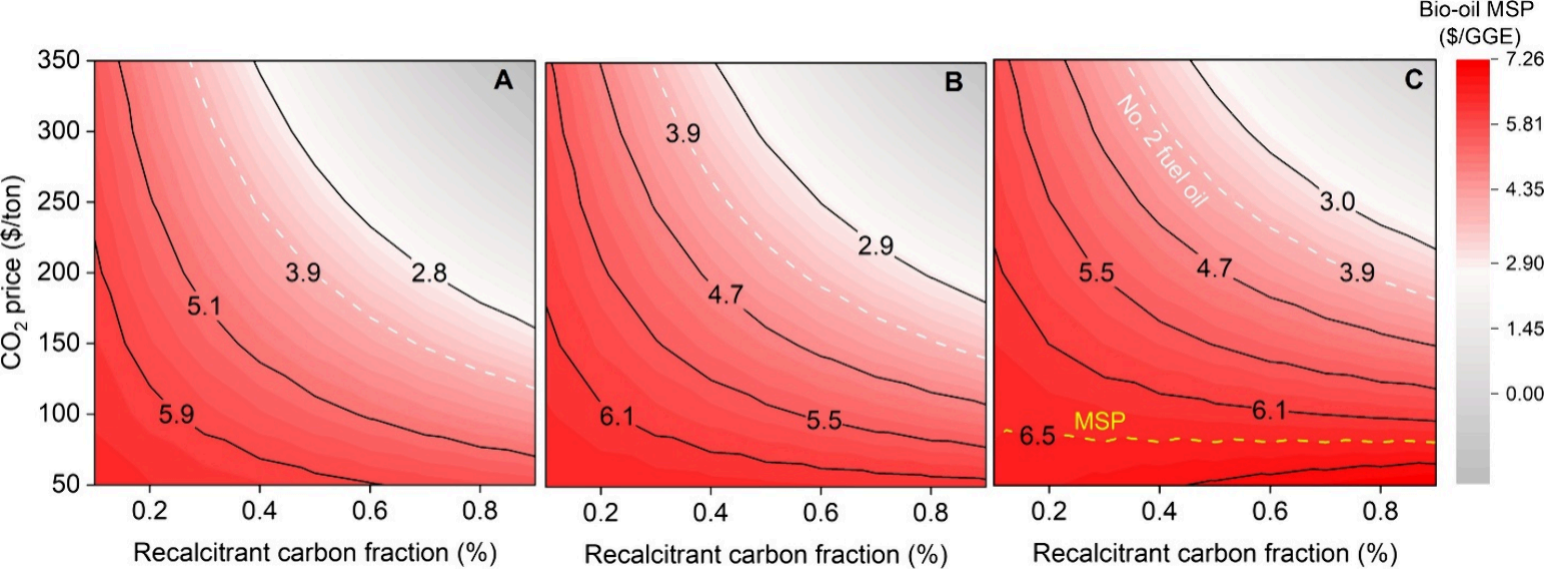
Contour plots illustrate the combined influence of recalcitrant carbon fraction, RCF (0.1–0.9) and CO_2_ price ($50–350/tCO_2_) on bio-oil MSP ($/GGE) under three Measurement, Monitoring, Reporting, and Verification (MMRV) and biochar transportation cost scenarios. (A) $12/tCO_2_ and $5/t, (B) $20/tCO_2_ and $20/t, and (C) $40/tCO_2_ and $40/t, respectively. Regions shaded in red indicate higher MSP, while gray zones represent cost-parity or negative-MSP cases, where carbon credit revenues offset production costs. Increasing recalcitrant carbon fraction (RCF) and CO_2_ price lowers MSP, whereas higher MRV and transport costs shift parity regions upward, underscoring their impact on carbon credit valuation and economic feasibility. Data for this figure are provided in Table S30.

Across all scenarios, higher RCF and CO_2_ prices significantly lower the MSP, indicating greater economic benefit when biochar’s carbon permanence and carbon credit value are maximized. Under the low-cost MMRV and transport scenario ($12/t and $5/t; Figure 5A), bio-oil MSP approaches cost parity with conventional fuel at RCF ≥ 70% and CO_2_ prices above $200/t. Increasing MMRV and transport costs (Figure 5B,C) shifts the parity region upward, emphasizing the critical role of verification and logistics expenses in determining the net value of carbon sequestration credits. The gray-shaded regions in each plot denote favorable conditions, where CC revenues offset or eliminate the production cost of bio-oil.

## Discussion

### Feedstock Supply Chain Challenges

Feedstock logistics remain a key cost barrier to the at-scale production of energy and value-added products from agricultural and forest residues due to their dispersed nature, low bulk density (∼80–200 kg/m^3^ compared to >600 kg/m^3^ for coal), and high moisture content (30–60%) of the biomass feedstock.^1^ Ensuring a reliable supply requires coordinated sourcing from multiple streams such as forest thinnings, sawmill residues, agricultural residues supported by aggregator networks, and long-term contracts with growers. Key challenges include high collection and transportation costs, storage-related degradation losses, and the lack of large-scale infrastructure for forest residues, agricultural residues, or organic wastes. These challenges are reflected in the base-case feedstock cost assumed in the TEA and underscore the economic sensitivity to logistics parameters as high cost can drive the MSP up to 33% (Figure 3). Strategies to mitigate these barriers include localized preprocessing (e.g., pelletization, briquetting, torrefaction) to improve energy density and reduce transportation and storage costs, establishing plantation-based supply systems for consistent feedstock availability , and leveraging port- or barge-based transport to expand sourcing radii while reducing costs per ton-km.^82^ Equally important are siting decisions, where locating facilities near biomass-dense regions and investing in decentralized logistics infrastructure are key to minimizing supply risk. Additionally, aligning preprocessing with the specific requirements of conversion technologies (e.g., particle size, moisture, ash content) is critical to lowering costs and ensuring year-round operability.^83^

### Impact of Pyrolysis Conditions on Biochar Quality

The value and functional application of biochar are primarily governed by its physicochemical properties.^84^ Biochar derived from woody biomass is characterized by high fixed carbon and total carbon content, coupled with low ash content, and lower H/C and O/C molar ratios (Figure 4A,B, Table S2). These properties impart high recalcitrance to degradation, thereby enhancing persistence in soils and suitability for long-term carbon sequestration and sorption-based applications like wastewater treatment.^85^ Conversely, herbaceous (agricultural) residues or organic wastes are high in ash content and when pyrolyzed resist volatilization, therefore result in higher solids yield.^86^ The dominant process variables governing biochar physicochemical properties include pyrolysis temperature, residence time, heating rate, and intrinsic feedstock composition. For example, high temperature (*T*_pyr_ = 500–650 °C) typically decreases char yield^87^ but concurrently increase the surface area and porosity, particularly in woody biomass due to its high lignin content provides more structural rigidity than corn stover or food wastes.^88^ With respect to long-term stability, the atomic H/C and O/C molar ratios serve as critical proxies for aromatic condensation, carbonization degree, and oxidative resistance and thus are widely adopted as indicators of biochar’s environmental durability. Empirical evidence demonstrates that fast pyrolysis of woody biomass consistently yields biochars with the lowest H/C and O/C ratios (typically ≤ 0.6 and ≤ 0.2, respectively), corroborating prior findings by Jalali et al.^85^ underscoring the superior recalcitrance and durability of fast pyrolysis biochars (Figure 4A,B) in the present analysis.

### Biochar for Supporting Data Center Power Loads

Future data centers especially those supporting large-scale generative AI systems will demand exponentially higher power, with energy intensities projected up to 40 times greater than traditional office buildings and cooling requirements reaching 40% of total energy use.^89^ In 2023, the U.S. alone consumed 4.4% of total national electricity and emitted about 105 MMT of carbon emissions.^90^ Meeting these huge energy demands sustainably requires leveraging diverse renewable energy resources because this will place significant pressure on existing renewable technologies.^91^ Although wind and solar are often prioritized for their efficiency and cost-competitiveness, their deployment is highly site-specific and constrained by temporal variability.^92^ For instance, in Michigan, where average wind speeds are only 7–8 m/s, energy systems analyses indicate that torrefied biomass from locally abundant feedstocks (e.g., Poplar) provides a more practical renewable option, particularly given the state’s existing coal-fired infrastructure that can be cofired or retrofitted for torrefied biomass combustion.^93^ Peak operational hours of data centers may not coincide with periods of optimal solar or wind availabilities, and the majority of data center hubs are located in urban areas or near technology clusters such as Silicon Valley, where local access to these intermittent resources is limited, and long-distance transmission could introduce significant losses. Regions with abundant woody or agricultural residues such as southeastern, Pacific Northwest, and Midwest can leverage biochar, such as that produced as a byproduct of pyrolysis, in cofiring or retrofitted power plants, providing both electricity and thermal energy while sequestering carbon.^1,2^ Unlike solar and wind, biochar-derived energy is not constrained by diurnal or weather variability, making it a reliable option for high-demand data center facilities that require 24/7 operation with minimal downtime and resilient grid independence. Additionally, biochar can be incorporated into the substantial material mass used in constructing data centers, serving as a carbon reservoir to store CO_2_ and offset emissions.^28^

### Emerging High-Value Applications of Biochar

The lowest-cost and lowest-risk application of biochar is as a soil amendment, where its intrinsic properties such as porosity, surface area, charge etc. enhance soil microbial activity, increase nutrient availability (N,P,K), boost soil microbial biomass, increase crop yield (7–30%)^94^ and elevate total soil organic carbon relative to control conditions. However, these effects are highly variable, depending on factors such as soil and crop type as well as pyrolysis conditions. Biochar from waste such as sewage sludge may contain toxic metals that can limit land application due to food chain contamination, while FP biochars often exhibit a high degree of aromatic condensation and may contain persistent PAHs that are recalcitrant and exert toxic effects on soil microbiota.^95^ Consequently, production costs for biochar are highly variable, ranging from $100–500/t,^33,34,66,82^ with the present study reporting $188–260/t (Figure 2C). To enable more consistent performance and higher-value applications, biochar can be engineered to have high surface area and pore structure and improved elemental composition. Beyond soil amendment, tailored biochars have potential in premium markets with significantly higher value. For instance, they can partially (50%) or fully (100%) substitute metallurgical coke, enabling 4–93% CO_2_ reduction in ironmaking.^66,96^ In cement and concrete applications, biochar can serve as an additive to lower process emissions in a sector responsible for ∼7% of global greenhouse gas (GHG) emissions.^97^ Biochar holds value in VCMs, where credits are prices between $1–150/ton_CO2_,^27,98^ and in environmental remediation, where activated carbon is applied for wastewater pollutant removal with reported costs ranging from $340–2,200/t.^34^ Lastly, biochar can be integrated into forest management strategies to mitigate wildfire risks by lowering surface fuel loads.

### Techno-Economic Considerations for Biochar Facilities

The economic and environmental feasibility of biomass biorefineries depend on multiple factors, including feedstock type and price, target products and coproducts, and plant scale. In this study, biochar and bio-oil were selected as principal products for the discounted cash flow rate-of-return analysis, which estimated the MSP while accounting credits from coproducts sales. Principal product selection is typically guided by either maximum yield or revenue potential but can be adjusted to optimize profitability and/or minimize GHG emissions.^99^ Commercial scale biorefineries (≥400 metric tpd) should operate in a phased manner, beginning with demonstration-scale operations (10–50 metric tpd) to explore high value applications of products, validate technical performance, and mitigate technical and market risks, especially for new products if the bioeconomy aims to diversify their business strategy.^100^ Additionally, securing long-term contracts for feedstock supply and biochar/bio-oil offtake is critical for financial stability and operational continuity, ensuring predictable cash flows and supporting sustainable, large-scale deployment.^101^

## Conclusions

This study provides a process-level techno-economic assessment of slow and fast pyrolysis pathways for converting woody biomass, agricultural residues, and organic wastes into biochar, bio-oil, and gaseous coproducts. Results indicate that biochar production via slow pyrolysis can already achieve cost parity with market values, particularly when coupled with soil amendment or emerging carbon credit markets. Fast pyrolysis bio-oil, while currently more expensive than fossil-derived fuels, demonstrates potential for cost reduction through economies of scale, carbon crediting, policy incentives, and high-value applications of coproduced biochar. Importantly, although fast pyrolysis currently implies less favorable economics than slow pyrolysis, it produces biochar of superior quality, characterized by lower H/C and higher recalcitrance, making it more suitable for durable carbon sequestration and premium applications such as industrial materials or cofiring. Sensitivity analysis highlighted feedstock cost, product yield, and plant capacity as key levers for improving economic performance, while scenario modeling illustrates how emerging voluntary carbon markets could accelerate deployment. Taken together, these findings emphasize the dual role of biomass pyrolysis systems in supplying alternate fuels and supporting low-carbon energy and bioproducts while also underscoring the need for continued innovation in feedstock logistics, process integration, and carbon valorization frameworks to enable large-scale deployment.

## Supplementary Material



## Notes

Additional details on fast and slow pyrolysis product yields capital and operating expenditures for additional feedstock cases (corn stover and food waste); Techno-economic analysis assumptions, including financial parameters, direct and indirect costs, labor and maintenance estimates; Minimum selling price breakdown; Sensitivity analysis parameters, rationale, and data for the tornado chart, and the methodologies used for Figures 4C and 5 (PDF)
