# Supplementary material for: Process Feasibility Analysis of Waste Biomass Valorization to Biochar and Bio-Oil via Slow and Fast Pyrolysis
Source: Energy Fuels. 2025 Dec 25;40(9):4670–82. doi: 10.1021/acs.energyfuels.5c05655 (PMC12969265; doi:10.1021/acs.energyfuels.5c05655)
Supplement: Supplementary file 1 [file ef5c05655_si_001.pdf]

**Supporting Information for:**

# Process feasibility analysis of waste biomass valorization to biochar and bio-oil via slow and fast pyrolysis

Geetanjali Yadav,[1*] Patrick Lamers[2]

1. Catalytic Carbon Transformation and Scale-up Center, National Renewable Energy Laboratory, Golden CO 80401, USA
2. Strategic Energy Analysis Center, National Renewable Energy Laboratory, Golden CO 80401, USA

*Correspondence: Geetanjali.Yadav@nrel.gov

**Abbreviations:**

CAPEX: Capital Expenditures
CC: Carbon Credits
$CO_2$: Carbon Dioxide
CDR: Carbon Dioxide Removal
CS: Corn Stover
DCFROR: Discounted Cash Flow Rate of Return
EBC: European Biochar Certificate
FP: Fast Pyrolysis
FW: Food Waste
GGE: Gasoline-Gallons Equivalent
GHG: Greenhouse Gas
H/C: Hydrogen by Carbon Molar Ratio
IBI: International Biochar Initiative
IQR: Interquartile Range
IRR: Internal Rate of Return
ISBL: Inside Battery Limits
MMRV: Measurement, Monitoring, Reporting, and Verification
MMT: Million Metric Tons
MSP: Minimum Selling Price
MTPD: Metric Tons Per Day
NCG: Non-Condensable Gases
NPV: Net Present Value
O/C: Oxygen by Carbon Molar Ratio
OPEX: Operating Expenses
PAH: Polycyclic Aromatic Hydrocarbons
PW: Pine Wood
RCF: Recalcitrant Carbon Fraction
RFS2: Renewable Fuel Standard 2
RINs: Renewable Identification Numbers
SP: Slow Pyrolysis
TEA: Techno-Economic Analysis
TCI: Total Capital Investment
TIC: Total Installed Capital
VCM: Voluntary Carbon Market
U.S.: United States

**Supplemental Items**


| | |
|---|---|
| **Abbreviations** | 2 |
| **Part I - Supporting figures** | |
| **Figure S1** Yields of products and co-products for (A) Slow pyrolysis and (B) Fast pyrolysis of three representative biomass feedstocks, pine wood (PW), corn stover (CS)**,** and food waste (FW). | 5 |
| **Figure S2** (A) Capital expenditure (CAPEX) and (B) Annual operating **expense for corn stover in slow pyrolysis.** | 6 |
| **Figure S3** (A) CAPEX and (B) Annual operating expense for food **waste in slow pyrolysis.** | 6 |
| **Figure S4** (A) CAPEX and (B) annual operating expense for corn **stover in fast pyrolysis.** | 7 |
| **Figure S5** Capital and annual operating expense for food waste in fast pyrolysis.**Figure S5 Capital and annual operating expense for food waste in fast pyrolysis.** | 7 |
| | |
| **Part II - Supporting tables** | 8 |
| **Table S1** Financial parameters used in discounted cash flow rate of return (DCFROR) analysis. | 8 |
| **Table S2** Comparative properties of pyrolysis products from slow vs fast pyrolysis | 9 |
| **Table S3** CAPEX breakdown for the slow pyrolysis cases, related to Fig. 2A from main text and Fig S2A in the ESI. | 10 |
| **Table S4** CAPEX breakdown for the fast pyrolysis cases, related to Fig. 2D from main text and Fig S3A in the ESI.**Error! Reference source not found.** | 10 |
| **Table S5** Annual operating cost by process section for the biochar product using PW, related to Fig. 2B in the main text. | 11 |
| **Table S6** Annual operating cost by process section for the biochar product using CS, related to Fig. S2B in the ESI. | 11 |
| **Table S7** Annual operating cost by process section for the biochar product using FW, related to Fig. S3B in the ESI. | 12 |
| **Table S8** Annual operating cost by process section for the bio-oil product using PW, related to Fig. 2E in the main text. | 12 |
| **Table S9** Annual operating cost by process section for the bio-oil product using CS, related to Fig. S4B in the ESI. | 13 |
| **Table S10** Annual operating cost by process section for the bio-oil product using FW, related to Fig. S5B in the ESI. | 13 |
| **Table S11** Cost factors for indirect costs. | 14 |
| **Table S12** Salary cost for plant employees. | 14 |
| **Table S13** Fixed operating costs for PW, CS, and FW in slow pyrolysis case. | 15 |
| **Table S14** Fixed operating costs for PW, CS, and FW in fast pyrolysis case. | 16 |
| **Table S15** Operating costs and summary of variable operating cost additions. | 16 |

| | |
|---|---|
| **Table S16** Simplified breakdown of the MSP for biochar product in the slow pyrolysis, related to Fig 2C from main text | 17 |
| **Table S17** Simplified breakdown of the MSP for bio-oil product in the fast pyrolysis, related to Fig 2F from main text | 17 |
| **Table S18** Feedstock price for the base case, low and high-cost scenarios for the sensitivity scenarios | 17 |
| **Table S19** Rationale for choosing the low and high values for the univariate sensitivity analysis, related to Fig 3 in the main text. | 18-19 |
| **Table S19** Rationale for choosing the low and high values for the univariate sensitivity analysis, related to Fig 3 in the main text.**Table S20** Sensitivity results for change to MSP of biochar ($198/t) from PW, related to Fig. 3A in the main text**Table S18** Feedstock price for the base case, low and high-cost scenarios for the sensitivity scenarios | 20 |
| **Table S21** Sensitivity results for change to MSP of biochar ($188/t) from CS, related to Fig. 3B in the main text | 20 |
| **Table S22** Sensitivity results for change to MSP of biochar ($260/t) from FW, related to Fig. 3C in the main text | 21 |
| **Table S23** Sensitivity results for change to MSP of bio-oil ($6.49/GGE) from PW, related to Fig. 3D in the main text | 21 |
| **Table S24** Sensitivity results for change to MSP of bio-oil ($7.42/GGE) from CS, related to Fig. 3E in the main text | 22 |
| **Table S25** Sensitivity results for change to MSP of bio-oil ($9.68/GGE) from FW, related to Fig. 3F in the main text | 22 |
| | |
| **Part III – Supplemental methods and materials** | 23-30 |
| **Table S26** Economic assessment of biochar substitution in cement, assuming a biochar price of $90/t, carbon credit price of $50/t $CO_2$, and cement priced at $100/t.* | 23 |
| **Table S27** Bio-oil MSP ($/GGE) or cement blending with biochar at 5%, 25%, and 50% ratios across varying biochar, cement, and carbon credit prices. | 24 |
| **Table S28** Economic assessment of biochar substitution in metallurgical coke, assuming a biochar price of $90/t, carbon credit price of $50/t $CO_2$, and met coke priced at $130/t.* | 24 |
| **Table S29** Bio-oil MSP ($/GGE) for metallurgical coke blending with biochar at 5%, 25%, and 50% ratios across varying biochar, metallurgical coke, and carbon credit prices.**Table S28** Economic assessment of biochar substitution in metallurgical coke, assuming a biochar price of $90/t, carbon credit price of $50/t $CO_2$, and met coke priced at $130/t.* | 25 |
| **Table S30** Effect of MRV and transportation cost on the bio-oil MSP, related to Figure *5A-C* in the main text**Table S30** Effect of MRV and transportation cost on the bio-oil MSP, related to Figure 5A-C in the main text | 27-30 |
| | |
| **Supplemental references** | 31 |

## Supporting Figures

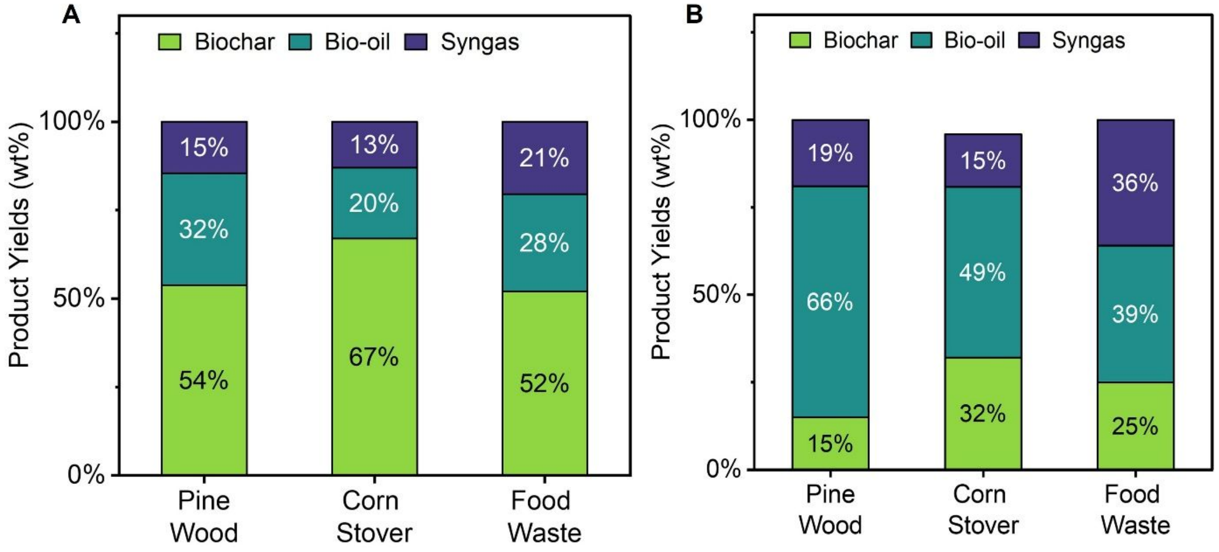


**Figure S1 Yields of products and co-products for (A) Slow pyrolysis and (B) Fast pyrolysis of three representative biomass feedstocks, pine wood (PW), corn stover (CS), and food waste (FW).**

In slow pyrolysis (~300°C, Panel A), biochar is the predominant product (52-67 wt%), with the highest yield from corn stover (67 wt%) due to its higher lignocellulosic content and lower volatile fraction. Bio-oil yields range from 20-32 wt%, while syngas represent the smallest fraction (13-21 wt%), reflecting limited secondary cracking at lower temperatures and longer residence times. In contrast, fast pyrolysis (500-600°C, Panel B) shifts the product distribution toward bio-oil (39–66 wt%) due to enhanced thermal decomposition and vapor-phase conversion of biomass volatiles. Biochar yields decrease to 15-32 wt% because of increased carbon volatilization, while syngas rise to 15-36 wt%, particularly for food waste, which contains higher moisture and labile organics favoring gas-phase reactions.

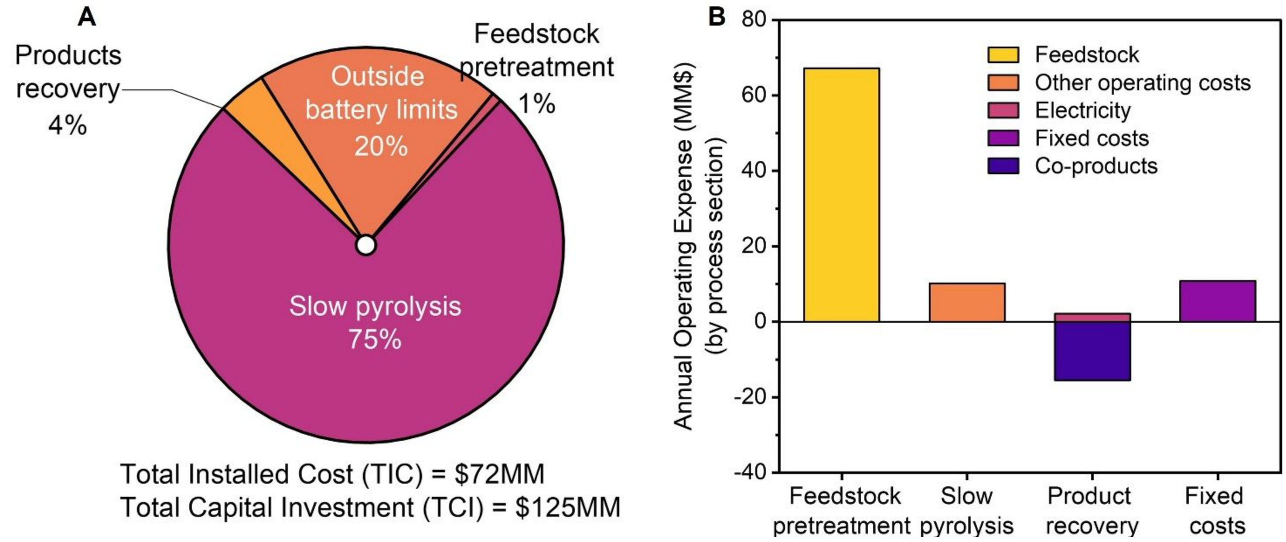


**Figure S2 (A) Capital expenditure (CAPEX) and (B) Annual operating expense for corn stover in slow pyrolysis.**

The TIC is $72MM and TCI is $125MM, primarily driven by the reactor system, feedstock preprocessing, and gas handling units, which together account for ~80% of total installed equipment cost. The total annual operating expense (OPEX) is $88MM, while the net OPEX, after accounting for co-product credits, is reduced to $73MM, dominated by feedstock supply and handling (~92%), followed by utilities and labor. Note: The positive y-axis values for the product recovery category are scaled by a factor of 10 for clarity

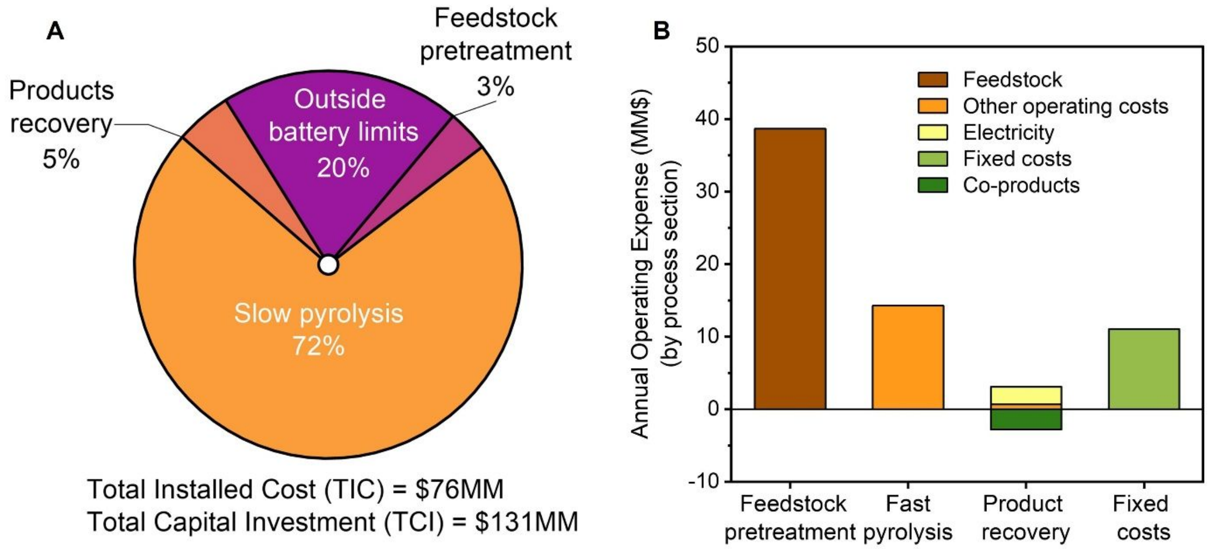


**Figure S3 (A) CAPEX and (B) Annual operating expense for food waste in slow pyrolysis.**

The TIC is $76MM and TCI is $131MM, primarily driven by the reactor system, feedstock preprocessing, and gas handling units. The gross OPEX is $64MM, while the net OPEX, after accounting for co-product credits, is reduced to $62MM. Note: The positive y-axis values for the product recovery category are scaled by a factor of 10 for clarity

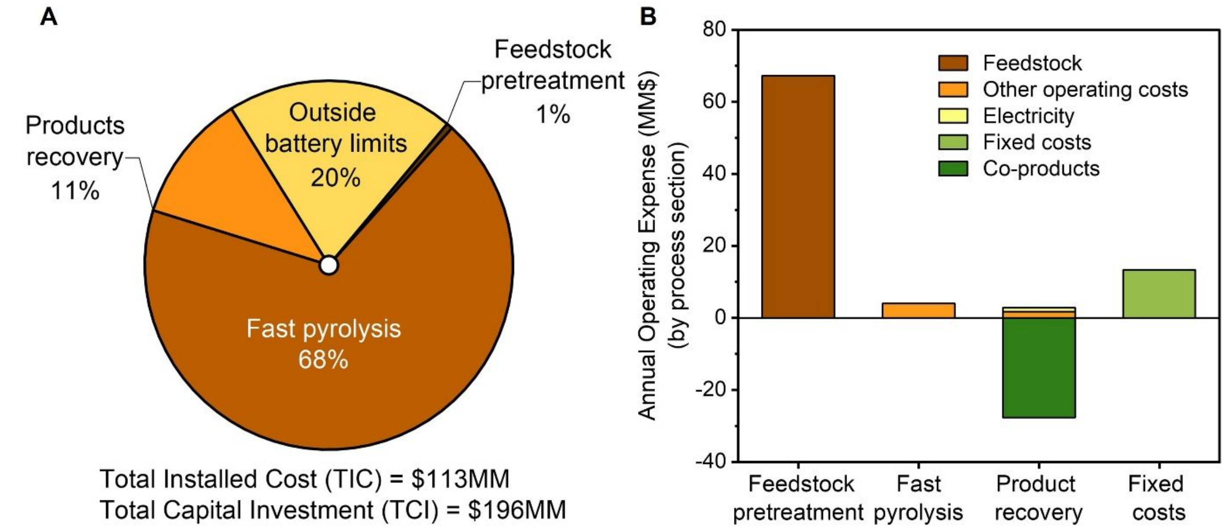


**Figure S4** (A) **CAPEX and (B) annual operating expense for corn stover in fast pyrolysis.**

The TIC is $113MM and TCI is $196MM, primarily driven by the reactor system, feedstock preprocessing, and gas handling units, which together account for ~80% of total installed equipment cost. The gross OPEX is $85MM, while the net OPEX, after accounting for co-product credits, is reduced to $59MM. Note: The positive y-axis values for the product recovery category are scaled by a factor of 10 for clarity

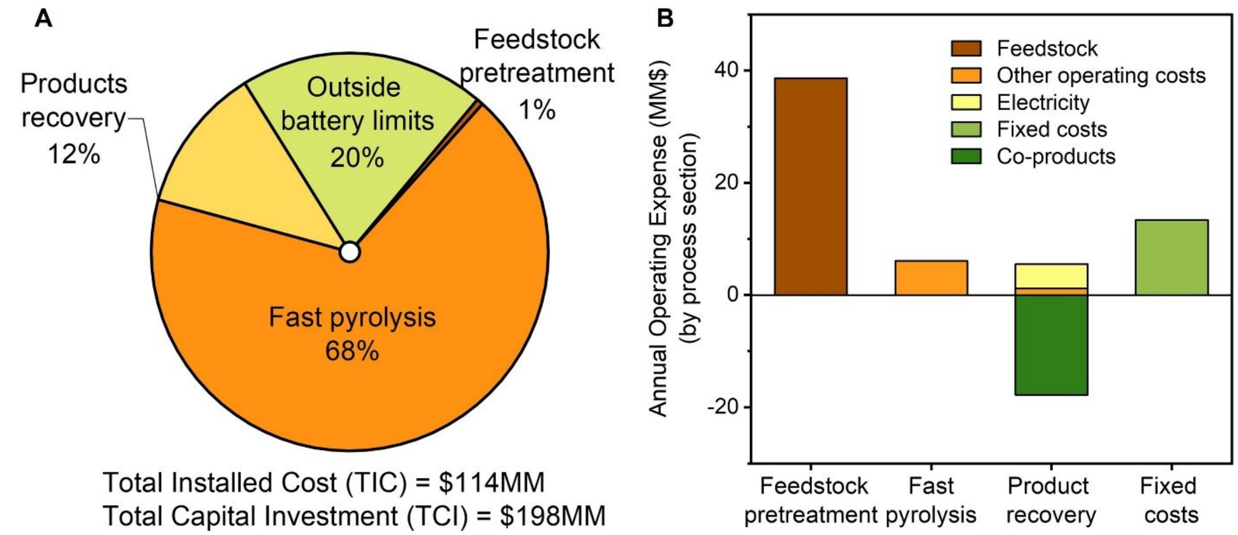


**Figure S5 Capital and annual operating expense for food waste in fast pyrolysis.**

The gross OPEX is $57MM, while the net OPEX, after accounting for co-product credits, is reduced to $40MM. Note: The positive y-axis values for the product recovery category are scaled by a factor of 10 for clarity

## B. Supporting Tables

**Table S1** Financial parameters used in discounted cash flow rate of return (DCFROR) analysis.

| **Discounted Cash Flow Financial Parameters** | | |
|---|---|---|
| Equity | (% of FCI) | 40% |
| Loan Interest | | 8.0% |
| Loan Term, years | | 10 |
| Working Capital | (% of FCI) | 5.0% |
| Discount Rate (IRR) | | 10% |
| Income Tax Rate | | 21% |
| Plant Depreciation Period | (Years) | 7 |
| Plant Life | (Years) | 30 |
| Construction Period | (Years) | 3 |
| % Spent in Year -2 | | 8% |
| % Spent in Year -1 | | 60% |
| % Spent in Year 0 | | 32% |
| Start-up Time | (Years) | 0.5 |
| Production year 1 | (% of Normal Capacity) | 50% |
| Variable Costs | (% of Normal) | 75% |
| Fixed Cost | (% of Normal) | 100% |
| Land Requirement | Acres | 10 |
| Land Cost | $/acre | $14,000 |

**Table S2** Comparative properties of pyrolysis products from slow vs fast pyrolysis reported in the literature

| Parameter | Unit | Slow Pyrolysis | Fast Pyrolysis | Remarks | References |
|---|---|---|---|---|---|
| **Biochar** | | | | | |
| Yield | wt% | High | Low | Longer residence time and lower temperatures favor secondary char-forming reactions, so more carbon stays in the solid phase. | [1] |
| O/C | molar | High | Low | Lower O/C indicates higher aromaticity and long-term stability. | [2,3] |
| H/C | molar | High | Low | Fast pyrolysis chars are more condensed | [2,3] |
| PAH content | % | Low at T< 550 C | High | Slow pyrolysis allows cracking/diffusion of PAHs; fast pyrolysis promotes PAH condensation during rapid devolatilization. | [4] |
| Nutrient retention | – | Higher | Lower | Slow pyrolysis biochars retain more K, Ca, Mg as it is not lost to vapors | [5] |
| Surface area | m²/g | 50–250 | 100-400 | Fast pyrolysis chars can be more porous due to rapid volatilization, though strongly feedstock- and temperature-dependent. | [5] |
| Soil suitability | – | Meets agronomic criteria | Typically, unsuitable without upgrading | Low H/C content makes FP biochar more stable but PAH content, pH, acidity makes it less suitable for soil amendment application unless upgraded. | [2,5,6] |
| **Bio-oil** | | | | | |
| Yield | wt% | 10-25 | 50-75 | Fast pyrolysis optimized for high oil yield | [7,8] |
| Bio-oil composition | wt% O | 20-30 | 45-50 | Rapid heating generates oxygen-rich vapors (sugars, aldehydes, phenols) that condense into a low-pH, thermally unstable liquid in fast pyrolysis | [9] |
| pH | – | 2.5-3.5 | 2.0-2.5 | Fast pyrolysis oils generally have more organic acids. | [5] |
| NCG composition | – | More $CO_2$/CO | More $CH_4$, $C_2$, $C_2$+ | Slow pyrolysis produces more CO/$CO_2$ from deoxygenation; fast pyrolysis yields more light hydrocarbons from thermal cracking. | [8] |

**Table S3** CAPEX breakdown for the slow pyrolysis cases, related to Fig. 2A from main text and Fig S2A in the ESI.

| **Process section** | **$MM** | **% TIC** | **$MM** | **% TIC** | **$MM** | **% TIC** |
|---|---|---|---|---|---|---|
| | **PW** | | **CS** | | **FW** | |
| Feedstock pretreatment | 0.6 | 0.8% | 0.6 | 0.8% | 2.6 | 3.5% |
| Slow pyrolysis | 54.3 | 74% | 54.4 | 75.2% | 54.3 | 71.8% |
| Product recovery | 3.8 | 5.2% | 2.9 | 4% | 3.5 | 4.7% |
| OSBL | 14.7 | 20% | 14.5 | 20% | 15.1 | 20% |
| **Total installed cost** | **73.4** | **100%** | **72.3%** | **100%** | **75.6** | **100%** |

**Table S4** CAPEX breakdown for the fast pyrolysis cases, related to Fig. 2D from main text and Fig S3A in the ESI.

| **Process section** | **$MM** | **% TIC** | **$MM** | **% TIC** | **$MM** | **% TIC** |
|---|---|---|---|---|---|---|
| | **PW** | | **CS** | | **FW** | |
| Feedstock pretreatment | 0.6 | 0.5% | 0.6 | 0.6% | 0.7 | 0.5% |
| Slow pyrolysis | 76.9 | 66.3% | 76.9 | 68.1% | 77.0 | 67.6% |
| Product recovery | 15.2 | 13.2% | 12.8 | 11.3% | 13.5 | 11.9% |
| OSBL | 23.2 | 20% | 22.6 | 20% | 22.8 | 20% |
| **Total installed cost** | **115.9** | **100%** | **112.9** | **100%** | **113.9** | **100%** |

**Table S5** Annual operating cost by process section for the biochar product using PW, related to Fig. 2B in the main text.

| | Yearly operating expenses breakdown ($MM/year) | | | | | |
|---|---|---|---|---|---|---|
| **Process area** | **Feedstock** | **Other operational costs** | **Electricity** | **Fixed costs** | **Co-products** | **Total** |
| Feedstock pretreatment | 68.68 | 0.00 | 0.00 | 0.00 | 0.00 | 68.86 |
| Slow pyrolysis | 0.00 | 5.06 | 0.00 | 0.00 | 0.00 | 5.06 |
| Product recovery | 0.00 | 0.00 | 0.28 | 0.00 | 0.00 | 0.28 |
| Co-products | 0.00 | 0.00 | 0.00 | 0.00 | -24.03 | -24.03 |
| Fixed costs | 0.00 | 0.00 | 0.00 | 10.89 | 0.00 | 10.89 |
| Total | 0.00 | 0.00 | 0.00 | 0.00 | 0.00 | 60.90# or 84.64* |

*# Total OPEX: with co-product credits*
**Total OPEX: without co-product credits*

**Table S6** Annual operating cost by process section for the biochar product using CS, related to Fig. S2B in the ESI.

| | Yearly operating expenses breakdown ($MM/year) | | | | | |
|---|---|---|---|---|---|---|
| **Process area** | **Feedstock** | **Other operational costs** | **Electricity** | **Fixed costs** | **Co-products** | **Total** |
| Feedstock pretreatment | 67.18 | 0.00 | 0.00 | 0.00 | 0.00 | 67.18 |
| Slow pyrolysis | 0.00 | 10.19 | 0.00 | 0.00 | 0.00 | 10.19 |
| Product recovery | 0.00 | 0.00 | 0.22 | 0.00 | 0.00 | 0.22 |
| Co-products | 0.00 | 0.00 | 0.00 | 0.00 | -15.50 | -15.50 |
| Fixed costs | 0.00 | 0.00 | 0.00 | 10.83 | 0.00 | 10.83 |
| Total | 0.00 | 0.00 | 0.00 | 0.00 | 0.00 | 72.93# or 88.21* |

*# Total OPEX: with co-product credits*
**Total OPEX: without co-product credits*

**Table S7** Annual operating cost by process section for the biochar product using FW, related to Fig. S3B in the ESI.

| | Yearly operating expenses breakdown ($MM/year) | | | | | |
|---|---|---|---|---|---|---|
| **Process area** | **Feedstock** | **Other operational costs** | **Electricity** | **Fixed costs** | **Co-products** | **Total** |
| Feedstock pretreatment | 38.65 | 0.00 | 0.00 | 0.00 | 0.00 | 38.65 |
| Slow pyrolysis | 0.00 | 14.28 | 0.00 | 0.00 | 0.00 | 14.28 |
| Product recovery | 0.00 | 0.66 | 0.24 | 0.00 | 0.00 | 0.91 |
| Co-products | 0.00 | 0.00 | 0.00 | 0.00 | -2.80 | -2.80 |
| Fixed costs | 0.00 | 0.00 | 0.00 | 11.03 | 0.00 | 11.03 |
| Total | 0.00 | 0.00 | 0.00 | 0.00 | 0.00 | 62.06[#] or 63.96[*] |

*# Total OPEX: with co-product credits*
**Total OPEX: without co-product credits*

**Table S8** Annual operating cost by process section for the bio-oil product using PW, related to Fig. 2E in the main text.

| | Yearly operating expenses breakdown ($MM/year) | | | | | |
|---|---|---|---|---|---|---|
| **Process area** | **Feedstock** | **Other operational costs** | **Electricity** | **Fixed costs** | **Co-products** | **Total** |
| Feedstock pretreatment | 62.61 | 0.00 | 0.00 | 0.00 | 0.00 | 62.61 |
| Slow pyrolysis | 0.00 | 4.69 | 0.00 | 0.00 | 0.00 | 4.69 |
| Product recovery | 0.00 | 0.44 | 0.17 | 0.00 | 0.00 | 0.61 |
| Co-products | 0.00 | 0.00 | 0.00 | 0.00 | -10.96 | -10.96 |
| Fixed costs | 0.00 | 0.00 | 0.00 | 13.49 | 0.00 | 13.49 |
| Total | 0.00 | 0.00 | 0.00 | 0.00 | 0.00 | 70.44[#] or 80.79[*] |

*# Total OPEX: with co-product credits*
**Total OPEX: without co-product credits*

**Table S9** Annual operating cost by process section for the bio-oil product using CS, related to Fig. S4B in the ESI.

| | **Yearly operating expenses breakdown ($MM/year)** | | | | | |
|---|---|---|---|---|---|---|
| **Process area** | **Feedstock** | **Other operational costs** | **Electricity** | **Fixed costs** | **Co-products** | **Total** |
| Feedstock pretreatment | 67.25 | 0.00 | 0.00 | 0.00 | 0.00 | 67.25 |
| Slow pyrolysis | 0.00 | 4.00 | 0.00 | 0.00 | 0.00 | 4.00 |
| Product recovery | 0.00 | 1.72 | 0.11 | 0.00 | 0.00 | 1.83 |
| Co-products | 0.00 | 0.00 | 0.00 | 0.00 | -27.70 | -27.70 |
| Fixed costs | 0.00 | 0.00 | 0.00 | 13.31 | 0.00 | 13.31 |
| Total | 0.00 | 0.00 | 0.00 | 0.00 | 0.00 | 58.69# or 84.55* |

*# Total OPEX: with co-product credits*
**Total OPEX: without co-product credits*

**Table S10** Annual operating cost by process section for the bio-oil product using FW, related to Fig. S5B in the ESI.

| | **Yearly Operating Expenses Breakdown ($M/year)** | | | | | |
|---|---|---|---|---|---|---|
| **Process area** | **Feedstock** | **Other Operational Costs** | **Electricity** | **Fixed Costs** | **Co-products** | **Total** |
| Feedstock pretreatment | 38.65 | 0.00 | 0.00 | 0.00 | 0.00 | 38.65 |
| Slow pyrolysis | 0.00 | 6.07 | 0.00 | 0.00 | 0.00 | 6.07 |
| Product recovery | 0.00 | 1.19 | 0.43 | 0.00 | 0.00 | 1.63 |
| Co-products | 0.00 | 0.00 | 0.00 | 0.00 | -17.79 | -17.79 |
| Fixed costs | 0.00 | 0.00 | 0.00 | 13.37 | 0.00 | 13.37 |
| Total | 0.00 | 0.00 | 0.00 | 0.00 | 0.00 | 41.92# or 58.09* |

*# Total OPEX: with co-product credits*
**Total OPEX: without co-product credits*

**Table S11** Cost factors for indirect costs.

| Indirect costs | % of TDC |
|---|---|
| Prorated expenses | 10.0 |
| Field expenses | 10.0 |
| Home office and construction fee | 20.0 |
| Project contingency | 10.0 |
| Other costs (start-ups, Permits, etc.) | 10.0 |
| **Total indirect costs** | **60.0** |

*Excluding land purchase cost

**Table S12** Salary cost for plant employees.

| Position | Salary (2016) | Number of positions | Total cost (2020) |
|---|---|---|---|
| Plant manager | 147,000 | 1 | 196,000 |
| Plant engineer | 70,000 | 1 | 94,000 |
| Maintenance supervisor | 57,000 | 1 | 76,000 |
| Maintenance technician | 40,000 | 16 | 853,000 |
| Lab manager | 56,000 | 1 | 75,000 |
| Laboratory technician | 40,000 | 2 | 107,000 |
| Shift supervisor | 48,000 | 5 | 320,000 |
| Shift operators | 40,000 | 20 | 1,066,000 |
| Yard employees | 28,000 | 12 | 448,000 |
| Clerks & secretaries | 36,000 | 3 | 144,000 |
| **Total salaries (2020$/yr)** | | | 3,379,000 |
| Labor burden | 90% of total salaries | | 3,379,000 |

Note: Labor costs are indexed, if necessary, to values from the U.S. Bureau of Labor Statistics (http://data.bls.gov/cgi-bin/srgate CEU3232500008).

**Table S13** Fixed operating costs for PW, CS, and FW in slow pyrolysis case.

| Cost item | Factor | Total cost for PW (2020) $ | Total cost for CS (2020) $ | Total cost for FW (2020) $ |
|---|---|---|---|---|
| Labor burden | 90% of total salaries | 3,379,000 | 3,379,000 | 3,379,000 |
| Overhead and benefits | 90% of labor and supervision | 3,073,000 | 3,037,000 | 3,037,000 |
| Maintenance | 3.0% of fixed capital investment (FCI*) | 3,636,000 | 3,584,000 | 3,746,000 |
| Property insurance and tax | 0.7% of (FCI*) | 849,000 | 837,000 | 875,000 |
| **Total fixed operating costs** | | **7,522,000** | **7,458,000** | **7,658,000** |

*Percentage of FCI excludes land purchase cost

**Table S14** Fixed operating costs for PW, CS, and FW in fast pyrolysis case.

| Cost item | Factor | Total cost for PW (2020) $ | Total cost for CS (2020) $ | Total cost for FW (2020) $ |
|---|---|---|---|---|
| Labor burden | 90% of total salaries | 3,379,000 | 3,379,000 | 3,379,000 |
| Overhead and benefits | 90% of labor and supervision | 3,073,000 | 3,037,000 | 3,037,000 |
| Maintenance | 3.0% of fixed capital investment (FCI*) | 5,742,000 | 5,594,000 | 5,642,000 |
| Property insurance and tax | 0.7% of (FCI*) | 1,340,000 | 1,306,000 | 1,317,000 |
| **Total fixed operating costs** | | **10,119,000** | **9,937,000** | **9,996,000** |

*Percentage of FCI excludes land purchase cost

**Table S15** Operating costs and summary of variable operating cost additions.

| Component | Cost (2016$) | Source |
|---|---|---|
| Steam | HP: $17.6/1000 kg,<br>MP: $15.3/1000 kg,<br>LP: $13.2/1000 kg | Seider *et al.* 2017 (Textbook) |
| Natural gas | $0.26/kg ($5/MMBtu) | Dutta *et al.* 2015 (Design Report) |
| Process water | $0.27/m$^3$ | Seider *et al.* 2017 (Textbook) |
| Cooling water | Based on volumetric flow | Calculated based on volumetric flow (m$^3$/s) as described in Ulrich and Vasudevan[4] |
| Electricity | $0.0572/kWh | Dutta *et al.* (Design Report). |

**Table S16** Simplified breakdown of the MSP for biochar product in the slow pyrolysis, related to Fig 2C from main text

| **Cost category** | **Cost contribution ($/t)** | | |
|---|---|---|---|
| | **PW** | **CS** | **FW** |
| Feedstock cost | 176.7 | 141.9 | 127.3 |
| Other operating cost | 13.8 | 22.0 | 43.7 |
| Co-product credits (Total)[1] | -61.8 | -32.7 | -9.2 |
| Capital charge | 41.3 | 34.0 | 61.9 |
| Fixed cost | 28.0 | 22.8 | 36.3 |
| **MSP** | **198.0** | **188.0** | **260.0** |

**Table S17** Simplified breakdown of the MSP for bio-oil product in the fast pyrolysis, related to Fig 2F from main text

| **Cost category** | **Cost contribution ($/GGE)** | | |
|---|---|---|---|
| | **PW** | **CS** | **FW** |
| Feedstock cost | 4.2 | 5.9 | 5.6 |
| Other operating cost | 0.4 | 0.6 | 1.1 |
| Co-product credits (Total)[1] | -0.7 | -2.5 | -2.6 |
| Capital charge | 1.7 | 2.2 | 3.7 |
| Fixed cost | 0.9 | 1.2 | 2.0 |
| **MSP** | **6.5** | **7.4** | **9.7** |

**Table S18** Feedstock price for the base case, low and high-cost scenarios for the sensitivity scenarios

| **Feedstocks** | **Low price ($/t)** | **Base case price ($/t)** | **High price ($/t)** |
|---|---|---|---|
| Pine Wood (PW) | 55 | 81 | 122 |
| Corn Stover (CS) | 82 | 87 | 122 |
| Food Waste (FW) | 30 | 50 | 70 |

[1] In the techno-economic analysis, the co-product is defined relative to the principal product: when biochar is the principal product (slow pyrolysis), bio-oil and NCGs are treated as co-products, and conversely, when bio-oil is the principal product (fast pyrolysis), biochar and NCGs are treated as the co-product.

**Table S19** Rationale for choosing the low and high values for the univariate sensitivity analysis, related to Fig 3 in the main text.

| **Sensitivity analysis parameter (Best case*: Base Case: Worst Case)** | **Justification for the high and low values of parameters** |
|---|---|
| Feedstock cost ($/MT)<br>55: **81**: 122 (Pine wood)<br>82: **87**: 122 (Corn stover)<br>30: **50**: 70 (Food waste) | **Best case:** Access to low- or negative-cost residues (e.g., sawmill or agri-waste) near site with minimal preprocessing and transport.<br>**Base case:** Average delivered cost for regional biomass/organic residues including handling and transport.<br>**Worst case:** Limited supply or competition increases haul distance and preprocessing needs. |
| Plant size (tpd)<br>1000: **2000**: 3000 | **Best case**: Modular scale-up or co-location strategies enable larger facilities benefiting from economies-of-scale.<br>**Base case**: Represents a typical commercial-scale thermochemical unit currently operating<br>**Worst case**: Demonstration-scale plants that do not leverage economies of scale. |
| Product yield (%)<br>+20: **base**: -20 (bio-oil case)<br>-20: base: +20 (biochar case) | **Best case**: Mature, low-risk technology with proven conversion performance.<br>**Base case**: Typical yield for medium-risk, benchmark TEA scenario.<br>**Worst case**: Early-stage technology with unoptimized conversion and product losses. |
| Reactor cost (x)<br>0.5×, **base**, 1.5× | **Best case**: Mature, modular reactor design leveraging prior demonstrations and vendor standardization; improved heat integration and material optimization reduce fabrication and installation costs.<br>**Base case**: Represents current commercial-scale reactor costs for thermochemical biomass conversion (e.g., pyrolysis) based on literature and cost-model benchmarks.<br>**Worst case**: Early-stage or custom-built reactor requiring specialized alloys, complex feeding systems, or additional gas cleanup units due to high ash or moisture content in feedstock. |
| Discount rate (%)<br>5: **10**: 15 | **Best:** Low-risk investment with mature technology, strong policy incentives, and established offtake agreements.<br>**Base:** Common value used for medium-risk TEA projects in renewable fuels and chemical sectors.<br>**Worst:** High-risk scenario with early-stage technology, volatile markets, or regulatory uncertainty. |
| Total capital investment factor (%)<br>(85, **100**, 130) | **Best case**: Energy integration leads to lower utilities usage causing a drop in operating expenses.<br>**Base case**: Standard separation approaches. |

| | |
|---|---|
| | **Worst case**: Reflects potential underestimation of auxiliary utilities to the plant. |
| Ash content (%)<br>0.9: **0.9**: 5 (Pine wood)<br>5.4: **5.4**: 8 (Corn stover)<br>8.8: **8.8**: 15 (Food waste) | **Best case**: same as base case as no additional pretreatment was carried out to remove ash content from the feedstocks.<br>**Base case**: Typical ash levels (3–5%) for agricultural residues such as corn stover or wheat straw, manageable with standard reactor design and periodic cleaning.<br>**Worst case**: High-ash feedstocks (≥8%) or contaminated residues increase maintenance and lowers efficiency. |
| Transportation cost (MM$)<br>0: **0**: +3 | **Best case**: Co-location of the biorefinery near major biomass sources (e.g., within 20–30 miles of feedstock supply hubs) minimizes hauling distance, fuel use, and logistics cost through optimized routing and backhauling strategies.<br>**Base case**: Typical transportation distance (50–75 miles) for dispersed agricultural or forest residues, representing standard trucking costs and handling fees used in regional biomass supply models.<br>**Worst case**: Sparse or seasonally variable feedstock availability requiring long-haul transport (>100 miles) or reliance on multiple small suppliers, increasing logistics complexity, fuel use, and delivered cost. |
| Income tax rate (%)<br>15: **21**: 35 | **Best:** Reduced corporate tax through credits, subsidies (e.g., Inflation Reduction Act), or operation in favorable tax jurisdictions.<br>**Base:** Standard U.S. federal corporate tax rate.<br>**Worst:** Includes potential additional state-level taxation or loss of credits/incentives. |

*Best case refers to parameters that result in lower MSP values

**Table S20** Sensitivity results for change to MSP of biochar ($198/t) from PW, related to Fig. 3A in the main text

| **Sensitivity analysis parameter (low cost: base case: high cost)** | **Change to MSP – Biochar (PW) ($/t)** | |
|---|---|---|
| | **Low cost** | **High cost** |
| Feedstock cost ($/t) 55: **81**: 122 | 142 | 288 |
| Plant size (tpd) 500: **240**: 100 | 183 | 233 |
| Biochar yield (%) 35: **54**: 75 | 180 | 232 |
| Reactor cost (MM$) 0.5× : **base**: 1.5× | 178 | 219 |
| Discount rate (%) 5: **10**: 15 | 184 | 213 |
| Total capital investment (%) 85: **100**: 130 | 191 | 214 |
| Ash content (%) 0.9: 0.9: 5 | 198 | 224 |
| Transportation cost (MM$) 0: **0**: 3 | 198 | 207 |
| Income tax rate (%) 15: **21**: 35 | 197 | 201 |

* Base case parameters are shown in bold at the center

**Table S21** Sensitivity results for change to MSP of biochar ($188/t) from CS, related to Fig. 3B in the main text

| **Sensitivity analysis parameter (low cost: base case: high cost)** | **Change to MSP – Biochar (PW) ($/t)** | |
|---|---|---|
| | **Low cost** | **High cost** |
| Feedstock cost ($/t) 82: **87**: 122 | 180 | 245 |
| Plant size (tpd) 500: **240**: 100 | 170 | 223 |
| Biochar yield (%) 35: **67**: 87 | 164 | 210 |
| Reactor cost (MM$) 0.5× : **base**: 1.5× | 171 | 205 |
| Discount rate (%) 5: **10**: 15 | 177 | 200 |
| Total capital investment (%) 85: **100**: 130 | 182 | 201 |
| Ash content (%) 5.4: **5.4**: 8 | 188 | 214 |
| Transportation cost (MM$) 0: **0**: 3 | 188 | 195 |
| Income tax rate (%) 15: **21**: 35 | 187 | 190 |

* Base case parameters are shown in bold at the center

**Table S22** Sensitivity results for change to MSP of biochar ($260/t) from FW, related to Fig. 3C in the main text

| Sensitivity analysis parameter (low cost: base case: high cost) | Change to MSP – Biochar (FW) ($/t) | |
|---|---|---|
| | Low cost | High cost |
| Feedstock cost ($/t) 30: **50**: 70 | 209 | 311 |
| Plant size (tpd) 500: **240**: 100 | 222 | 299 |
| Biochar yield (%) 35: **52**: 72 | 189 | 318 |
| Reactor cost (MM$) 0.5× : **base**: 1.5× | 234 | 286 |
| Discount rate (%) 5: **10**: 15 | 241 | 279 |
| Total capital investment (%) 85: **100**: 130 | 250 | 281 |
| Ash content (%) 8.8: **8.8**: 15 | 260 | 294 |
| Transportation cost (MM$) 0: **0**: 3 | 260 | 271 |
| Income tax rate (%) 15: **21**: 35 | 259 | 264 |

* Base case parameters are shown in bold at the center

**Table S23** Sensitivity results for change to MSP of bio-oil ($6.49/GGE) from PW, related to Fig. 3D in the main text

| Sensitivity analysis parameter (low cost: base case: high cost) | Change to MSP – Bio-oil (PW) ($/GGE) | |
|---|---|---|
| | Low cost | High cost |
| Feedstock cost ($/t) 55: **81**: 122 | 5.13 | 8.63 |
| Plant size (tpd) 500: **240**: 100 | 5.97 | 8.14 |
| Bio-oil yield (%) 80: **66**: 40 | 5.29 | 7.90 |
| Reactor cost (MM$) 0.5× : **base**: 1.5× | 6.12 | 6.86 |
| Discount rate (%) 5: **10**: 15 | 7.08 | 7.08 |
| Total capital investment (%) 85: **100**: 130 | 6.16 | 7.15 |
| Ash content (%) 0.9: 0.9: 5 | 6.49 | 6.93 |
| Transportation cost (MM$) 0: **0**: 3 | 6.49 | 6.71 |
| Income tax rate (%) 15: **21**: 35 | 6.45 | 6.61 |

* Base case parameters are shown in bold at the center

**Table S24** Sensitivity results for change to MSP of bio-oil ($7.42/GGE) from CS, related to Fig. 3E in the main text

| Sensitivity analysis parameter (low cost: base case: high cost) | Change to MSP – Bio-oil (CS) ($/GGE) | |
|---|---|---|
| | Low cost | High cost |
| Feedstock cost ($/t) 82: **87**: 122 | 5.23 | 9.82 |
| Plant size (tpd) 500: **240**: 100 | 6.33 | 9.42 |
| Bio-oil yield (%) 80: **49**: 40 | 5.36 | 9.22 |
| Reactor cost (MM$) 0.5× : **base**: 1.5× | 6.93 | 7.91 |
| Discount rate (%) 5: **10**: 15 | 6.69 | 8.18 |
| Total capital investment (%) 85: **100**: 130 | 7.00 | 8.26 |
| Ash content (%) 5.4: **5.4**: 8 | 6.94 | 7.91 |
| Transportation cost (MM$) 0: **0**: 3 | 7.42 | 7.71 |
| Income tax rate (%) 15: **21**: 35 | 7.37 | 7.57 |

* Base case parameters are shown in bold at the center

**Table S25** Sensitivity results for change to MSP of bio-oil ($9.68/GGE) from FW, related to Fig. 3F in the main text

| Sensitivity analysis parameter (low cost: base case: high cost) | Change to MSP – Bio-oil (FW) ($/GGE) | |
|---|---|---|
| | Low cost | High cost |
| Feedstock cost ($/t) 30: **50**: 70 | 7.45 | 11.91 |
| Plant size (tpd) 500: **240**: 100 | 8.51 | 11.84 |
| Bio-oil yield (%) 19: **39**: 59 | 8.29 | 11.28 |
| Reactor cost (MM$) 0.5× : **base**: 1.5× | 8.88 | 10.48 |
| Discount rate (%) 5: **10**: 15 | 8.47 | 10.93 |
| Total capital investment (%) 85: **100**: 130 | 8.99 | 11.06 |
| Ash content (%) 8.8: **8.8**: 15 | 9.68 | 10.82 |
| Transportation cost (MM$) 0: **0**: 3 | 9.68 | 10.15 |
| Income tax rate (%) 15: **21**: 35 | 9.59 | 9.92 |

* Base case parameters are shown in bold at the center

## A. Additional value proposition of biochar for cement additive and metallurgical coke substitution

The cement and steel industries are among the largest sources of $CO_2$, with high carbon intensity (CI) per unit of product. The CI of Portland cement is 0.8-0.95 metric ton (t) $CO_2$ per metric ton of cement. Similarly, metallurgical coke used in steelmaking has a CI of 2.8-3.0 $tCO_2$ per ton of coke. Reducing emissions in these sectors is critical to achieving global climate goals. Biochar, a carbon-rich product from biomass pyrolysis, offers the dual benefit of permanently storing carbon while substituting fossil-intensive materials. In cement, biochar can replace a portion of clinker, potentially reducing calcination-related $CO_2$ emissions whereas in metallurgical applications, it can substitute a portion of coke, lowering fossil carbon input and associated GHG emissions. To assess these benefits, biochar blending with conventional cement and metallurgical coke was analyzed at 5%, 25%, and 50% substitution levels, assuming equivalent material performance.

1. **Cement application:** This analysis quantified the potential revenue a biochar supplier could earn by selling biochar to a cement manufacturer, with the resulting credit used to offset the MSP of bio-oil produced from fast pyrolysis (**Table S26**). The base case biochar price was set at \$100/t, excluding any carbon credit considerations. For the cement blending application, biochar prices of \$90/t and \$350/t were considered to represent low and high market scenarios, respectively. Corresponding cement prices were assumed at \$100/t (low) and \$160/t (high). Carbon credit values associated with $CO_2$ sequestration in biochar were evaluated at two levels: \$50/$tCO_2$ and \$350/$tCO_2$, representing near-term and long-term carbon pricing scenarios. These parameters were combined to generate eight market scenarios (See **Table S27**) across three biochar blending levels: 5%, 25%, and 50%, assuming a fixed CI of 0.9 $tCO_2$ per ton of cement.

   For each case, the net economic benefit was calculated as the difference between the cement product price and the biochar-blended equivalent, accounting for the combined effects of material substitution and carbon credit revenue. It was further assumed that 75% of this revenue would be transferred to the biochar supplier, and this adjusted value was incorporated into the techno-economic analysis to estimate its contribution toward reducing the MSP of bio-oil.

**Table S26** Economic assessment of biochar substitution in cement, assuming a biochar price of \$90/t, carbon credit price of \$50/t $CO_2$, and cement priced at \$100/t.*

| Cement (ton) | Blend (%) | Biochar used (ton) | Adjusted cement cost | $CO_2$ saved (ton) | Carbon credit value | SCM credit | Net benefit per ton cement |
|---|---|---|---|---|---|---|---|
| 1.0 | 5% | 0.05 | \$5.00 | 0.045 | \$2.25 | \$2.50 | \$9.75 |
| 1.0 | 25% | 0.25 | \$25.00 | 0.225 | \$11.25 | \$12.50 | \$48.75 |
| 1.0 | 50% | 0.50 | \$50.00 | 0.45 | \$22.50 | \$25.00 | \$97.50 |

* Similar assessment was conducted for cement price at the high price of \$160/t.

**Table S27 B**io-oil MSP ($/GGE) or cement blending with biochar at 5%, 25%, and 50% ratios across varying biochar, cement, and carbon credit prices.

| Scenarios | Biochar price ($/t) | Cement price ($/t) | Carbon credit price ($/ton$CO_2$e) | MSP of bio-oil ($/GGE) for different biochar blend ratio | | |
|---|---|---|---|---|---|---|
| | | | | 5% | 25% | 50% |
| S1 | 90 | 100 | 50 | 6.51 | 6.29 | 6.02 |
| S2 | 90 | 100 | 350 | 6.35 | 5.50 | 4.44 |
| S3 | 350 | 100 | 50 | 4.58 | 4.37 | 4.10 |
| S4 | 350 | 100 | 350 | 4.43 | 3.58 | 2.51 |
| S5 | 90 | 160 | 50 | 6.49 | 6.21 | 5.86 |
| S6 | 90 | 160 | 350 | 6.34 | 5.42 | 4.27 |
| S7 | 350 | 160 | 50 | 4.57 | 4.28 | 3.93 |
| S8 | 350 | 160 | 350 | 4.41 | 3.49 | 2.35 |

2. **Metallurgical coke application:** A parallel analysis was conducted for metallurgical coke substitution in steelmaking, assuming biochar provides equivalent process performance (**Table S28**). Biochar prices of $90/t and $350/t and carbon credit values of $50/t$CO_2$ and $350/t$CO_2$ were applied to maintain consistency with the cement case. Metallurgical coke prices were assumed at $130/t (low) and $430/t (high), based on reported U.S. market data. These parameters were combined to form eight market scenarios (**Table S29**) across three biochar substitution levels: 5%, 25%, and 50%, assuming a fixed CI of 3.0 t$CO_2$ per ton of metallurgical coke. The net economic benefit was determined from the difference between the baseline coke price and the biochar-substituted equivalent, accounting for both material substitution and carbon credit revenues. As in the cement case, 75% of this revenue was assumed to be transferred to the biochar supplier and incorporated into the TEA to quantify its contribution to offsetting the bio-oil MSP.

**Table S28** Economic assessment of biochar substitution in metallurgical coke, assuming a biochar price of $90/t, carbon credit price of $50/t $CO_2$, and met coke priced at $130/t.*

| Met coke (ton) | Blend % | Biochar Used (ton) | Coke Cost Saved | $CO_2$ Saved (t) | Carbon Credit Value | Process Credit | Net benefit per ton coke |
|---|---|---|---|---|---|---|---|
| 1.0 | 5% | 0.05 | $21.50 | 0.15 | $7.50 | $1.50 | $30.50 |
| 1.0 | 25% | 0.25 | $107.50 | 0.75 | $37.50 | $7.50 | $152.50 |
| 1.0 | 50% | 0.50 | $215.00 | 1.50 | $75.00 | $15.00 | $305.00 |

* Similar assessment was conducted for met coke price at the high price of $430/t.

**Table S29** Bio-oil MSP ($/GGE) for metallurgical coke blending with biochar at 5%, 25%, and 50% ratios across varying biochar, metallurgical coke, and carbon credit prices.

| Scenarios | Biochar price ($/t) | Metallurgical coke price ($/t) | Carbon credit price ($/ton$CO_2$e) | MSP of bio-oil ($/GGE) for different biochar blend ratio | | |
|---|---|---|---|---|---|---|
| | | | | 5% | 25% | 50% |
| S1 | 90 | 130 | 50 | 6.48 | 6.13 | 5.70 |
| S2 | 90 | 130 | 350 | 6.23 | 4.88 | 3.20 |
| S3 | 350 | 130 | 50 | 4.55 | 4.21 | 3.78 |
| S4 | 350 | 130 | 350 | 4.30 | 2.96 | 1.28 |
| S5 | 90 | 430 | 50 | 6.40 | 5.72 | 4.87 |
| S6 | 90 | 430 | 350 | 6.15 | 4.47 | 2.37 |
| S7 | 350 | 430 | 50 | 4.47 | 3.79 | 2.94 |
| S8 | 350 | 430 | 350 | 4.22 | 2.54 | 0.45 |

## B. Effect of $CO_2$ price, RCF, MRV and biochar transportation costs on bio-oil MSP

A multivariate sensitivity analysis (**Figure 5A-C** in the main text) was performed that integrates the economic effects of carbon sequestration value, Monitoring, Reporting, and Verification (MRV) costs, and biochar transportation costs to provide a realistic estimate of bio-oil competitiveness under various carbon market conditions. Although base case bio-oil MSP of $6.49/GGE does not include any credit owing to biochar's carbon capture potential, this analysis provides an evolving futuristic carbon market scenario. Thus, two primary parameters were selected as follows:

1. **Parameter selection and rationale:**
   a. **$CO_2$ price ($/t$CO_2$):** Carbon prices are currently below the levels needed to meet climate targets, with 2030 projections suggesting an average of ~$85/t$CO_2$ to align with Paris Agreement goals.[10] Durable carbon removal credits, such as BiCRS and BECCS, command higher prices ($15-400/t$CO_2$) due to their permanence and verifiability.[11] Accordingly, a $CO_2$ price range of $50–350/t$CO_2$ was applied in this analysis to capture near-term policy baselines (2030) and long-term carbon-neutrality scenarios (2050).
   b. **Recalcitrant carbon fraction (RCF, %):** refers to the fraction of carbon in the produced biochar that is sufficiently stable (i.e. recalcitrant) to persist over long timescales (hundreds to thousands of years), rather than being rapidly decomposed or oxidized, varied from 0.1 to 0.9 (10-90%).[2] Because this carbon permanence underlies the value of biochar as a carbon-sequestration agent, the RCF is critical in calculating the carbon credit revenue.
      i. RCF was linked to the biochar's O/C atomic ratio, where $O/C < 0.2$ corresponds to $RCF \geq 80\%$ and $O/C > 0.6$ corresponds to $RCF \leq 50\%$, consistent with stability classifications reported in the literature.[2]

c. **MRV costs ($/t$CO_2$e):** included to represent expenses associated with carbon credit verification, ranging $12-40/t$CO_2$, based on published costs for direct air capture (DAC) and enhanced rock weathering (ERW) systems.[12,13] Since, the MRV costs for biochar, BiCRS or BECCS are not explicitly available, we included a range from $12-40/t$CO_2$e for this analysis.

d. **Transportation costs ($/t biochar):** Accounted for biochar logistics and regional storage/handling expenses, ranging $5-40/t$CO_2$ depending on distance to carbon utilization or storage sites.[12,13] Since, the biochar transportation costs for biochar, BiCRS or BECCS are not explicitly available, we included a range from $5-40/t$CO_2$e for this analysis.

2. **Modeling framework:**

The analysis builds on the techno-economic model for fast pyrolysis developed in Aspen Plus and integrated with post-processing routines in Microsoft Excel spreadsheet. The MSP of bio-oil was determined using a discounted cash flow rate of return (DCFROR) model with the following key features:

- **Base case:** 10-year plant life, 10% IRR, 90% capacity factor.
- **Feedstock:** Woody biomass with representative ultimate/proximate composition from literature.
- **Product slate:** Bio-oil, non-condensable gases, and biochar.
- **Biochar carbon credit revenue (CC):**

$$\text{CC Revenue} = (\text{CO}_2\text{ Price} - \text{MRV Cost} - \text{Transport Cost}) \times \text{RCF} \times \text{Biochar-C Yield}$$

where Biochar-C Yield (t$CO_2$ equivalent per ton biomass) was derived from modeled carbon distributions.

3. **Computational Procedure**

For each combination of $CO_2$ price, RCF, MRV cost, and transport cost, the net MSP of bio-oil ($/GGE) was recalculated by subtracting the carbon credit revenue from the total annualized production cost:

$$\text{MSP} = \frac{\text{Annualized Cost} - \text{CC Revenue}}{\text{Annual Bio-oil Production}}$$

Contour plots (**Figure 6A–C** in the main text) were generated to visualize the sensitivity of bio-oil MSP to $CO_2$ price and RCF under three cost scenarios:

(**A**) Low-cost MRV and transport ($12/t and $5/t)
**(B)** Moderate cost ($25/t and $20/t)
**(C)** High cost ($40/t and $40/t)

**Table S30** provide additional data used to plot **Figure 6A-C** in the main text.

**Table S30** Effect of MRV and transportation cost on the bio-oil MSP, related to Figure 5A-C in the main text

| RCF | $CO_2$ price | MRV cost | Transport cost | Bio-oil MSP | RCF | $CO_2$ price | MRV cost | Transport cost | Bio-oil MSP | RCF | $CO_2$ price | MRV cost | Transport cost | Bio-oil MSP |
|---|---|---|---|---|---|---|---|---|---|---|---|---|---|---|
| 90% | 50 | 12 | 5 | 5.6 | 90% | 50 | 20 | 20 | 6.2 | 90% | 50 | 40 | 40 | 7.3 |
| 80% | 50 | 12 | 5 | 5.7 | 80% | 50 | 20 | 20 | 6.3 | 80% | 50 | 40 | 40 | 7.2 |
| 70% | 50 | 12 | 5 | 5.8 | 70% | 50 | 20 | 20 | 6.3 | 70% | 50 | 40 | 40 | 7.1 |
| 60% | 50 | 12 | 5 | 5.9 | 60% | 50 | 20 | 20 | 6.3 | 60% | 50 | 40 | 40 | 7.0 |
| 50% | 50 | 12 | 5 | 6.0 | 50% | 50 | 20 | 20 | 6.3 | 50% | 50 | 40 | 40 | 6.9 |
| 40% | 50 | 12 | 5 | 6.1 | 40% | 50 | 20 | 20 | 6.4 | 40% | 50 | 40 | 40 | 6.8 |
| 30% | 50 | 12 | 5 | 6.2 | 30% | 50 | 20 | 20 | 6.4 | 30% | 50 | 40 | 40 | 6.7 |
| 20% | 50 | 12 | 5 | 6.3 | 20% | 50 | 20 | 20 | 6.4 | 20% | 50 | 40 | 40 | 6.7 |
| 10% | 50 | 12 | 5 | 6.4 | 10% | 50 | 20 | 20 | 6.5 | 10% | 50 | 40 | 40 | 6.6 |
| 90% | 100 | 12 | 5 | 4.4 | 90% | 100 | 20 | 20 | 5.0 | 90% | 100 | 40 | 40 | 6.0 |
| 80% | 100 | 12 | 5 | 4.6 | 80% | 100 | 20 | 20 | 5.1 | 80% | 100 | 40 | 40 | 6.0 |
| 70% | 100 | 12 | 5 | 4.8 | 70% | 100 | 20 | 20 | 5.3 | 70% | 100 | 40 | 40 | 6.1 |
| 60% | 100 | 12 | 5 | 5.1 | 60% | 100 | 20 | 20 | 5.5 | 60% | 100 | 40 | 40 | 6.1 |
| 50% | 100 | 12 | 5 | 5.3 | 50% | 100 | 20 | 20 | 5.6 | 50% | 100 | 40 | 40 | 6.2 |
| 40% | 100 | 12 | 5 | 5.5 | 40% | 100 | 20 | 20 | 5.8 | 40% | 100 | 40 | 40 | 6.3 |
| 30% | 100 | 12 | 5 | 5.8 | 30% | 100 | 20 | 20 | 6.0 | 30% | 100 | 40 | 40 | 6.3 |
| 20% | 100 | 12 | 5 | 6.0 | 20% | 100 | 20 | 20 | 6.1 | 20% | 100 | 40 | 40 | 6.4 |
| 10% | 100 | 12 | 5 | 6.3 | 10% | 100 | 20 | 20 | 6.3 | 10% | 100 | 40 | 40 | 6.4 |
| 90% | 150 | 12 | 5 | 3.1 | 90% | 150 | 20 | 20 | 3.7 | 90% | 150 | 40 | 40 | 4.7 |
| 80% | 150 | 12 | 5 | 3.5 | 80% | 150 | 20 | 20 | 4.0 | 80% | 150 | 40 | 40 | 4.9 |
| 70% | 150 | 12 | 5 | 3.8 | 70% | 150 | 20 | 20 | 4.3 | 70% | 150 | 40 | 40 | 5.1 |
| 60% | 150 | 12 | 5 | 4.2 | 60% | 150 | 20 | 20 | 4.6 | 60% | 150 | 40 | 40 | 5.3 |

| | | | | | | | | | | | | | | |
|---|---|---|---|---|---|---|---|---|---|---|---|---|---|---|
| 50% | 150 | 12 | 5 | 4.6 | 50% | 150 | 20 | 20 | 4.9 | 50% | 150 | 40 | 40 | 5.5 |
| 40% | 150 | 12 | 5 | 5.0 | 40% | 150 | 20 | 20 | 5.2 | 40% | 150 | 40 | 40 | 5.7 |
| 30% | 150 | 12 | 5 | 5.4 | 30% | 150 | 20 | 20 | 5.6 | 30% | 150 | 40 | 40 | 5.9 |
| 20% | 150 | 12 | 5 | 5.7 | 20% | 150 | 20 | 20 | 5.9 | 20% | 150 | 40 | 40 | 6.1 |
| 10% | 150 | 12 | 5 | 6.1 | 10% | 150 | 20 | 20 | 6.2 | 10% | 150 | 40 | 40 | 6.3 |
| 90% | 200 | 12 | 5 | 1.8 | 90% | 200 | 20 | 20 | 2.4 | 90% | 200 | 40 | 40 | 3.4 |
| 80% | 200 | 12 | 5 | 2.3 | 80% | 200 | 20 | 20 | 2.9 | 80% | 200 | 40 | 40 | 3.8 |
| 70% | 200 | 12 | 5 | 2.9 | 70% | 200 | 20 | 20 | 3.3 | 70% | 200 | 40 | 40 | 4.1 |
| 60% | 200 | 12 | 5 | 3.4 | 60% | 200 | 20 | 20 | 3.8 | 60% | 200 | 40 | 40 | 4.4 |
| 50% | 200 | 12 | 5 | 3.9 | 50% | 200 | 20 | 20 | 4.2 | 50% | 200 | 40 | 40 | 4.8 |
| 40% | 200 | 12 | 5 | 4.4 | 40% | 200 | 20 | 20 | 4.7 | 40% | 200 | 40 | 40 | 5.1 |
| 30% | 200 | 12 | 5 | 4.9 | 30% | 200 | 20 | 20 | 5.1 | 30% | 200 | 40 | 40 | 5.5 |
| 20% | 200 | 12 | 5 | 5.5 | 20% | 200 | 20 | 20 | 5.6 | 20% | 200 | 40 | 40 | 5.8 |
| 10% | 200 | 12 | 5 | 6.0 | 10% | 200 | 20 | 20 | 6.0 | 10% | 200 | 40 | 40 | 6.1 |
| 90% | 250 | 12 | 5 | 0.5 | 90% | 250 | 20 | 20 | 1.1 | 90% | 250 | 40 | 40 | 2.1 |
| 80% | 250 | 12 | 5 | 1.2 | 80% | 250 | 20 | 20 | 1.7 | 80% | 250 | 40 | 40 | 2.6 |
| 70% | 250 | 12 | 5 | 1.9 | 70% | 250 | 20 | 20 | 2.3 | 70% | 250 | 40 | 40 | 3.1 |
| 60% | 250 | 12 | 5 | 2.5 | 60% | 250 | 20 | 20 | 2.9 | 60% | 250 | 40 | 40 | 3.6 |
| 50% | 250 | 12 | 5 | 3.2 | 50% | 250 | 20 | 20 | 3.5 | 50% | 250 | 40 | 40 | 4.1 |
| 40% | 250 | 12 | 5 | 3.8 | 40% | 250 | 20 | 20 | 4.1 | 40% | 250 | 40 | 40 | 4.6 |
| 30% | 250 | 12 | 5 | 4.5 | 30% | 250 | 20 | 20 | 4.7 | 30% | 250 | 40 | 40 | 5.0 |
| 20% | 250 | 12 | 5 | 5.2 | 20% | 250 | 20 | 20 | 5.3 | 20% | 250 | 40 | 40 | 5.5 |
| 10% | 250 | 12 | 5 | 5.8 | 10% | 250 | 20 | 20 | 5.9 | 10% | 250 | 40 | 40 | 6.0 |
| 90% | 300 | 12 | 5 | -0.7 | 90% | 300 | 20 | 20 | -0.2 | 90% | 300 | 40 | 40 | 0.9 |
| 80% | 300 | 12 | 5 | 0.1 | 80% | 300 | 20 | 20 | 0.6 | 80% | 300 | 40 | 40 | 1.5 |
| 70% | 300 | 12 | 5 | 0.9 | 70% | 300 | 20 | 20 | 1.3 | 70% | 300 | 40 | 40 | 2.1 |

| | | | | | | | | | | | | | | |
|---|---|---|---|---|---|---|---|---|---|---|---|---|---|---|
| 60% | 300 | 12 | 5 | 1.7 | 60% | 300 | 20 | 20 | 2.1 | 60% | 300 | 40 | 40 | 2.7 |
| 50% | 300 | 12 | 5 | 2.5 | 50% | 300 | 20 | 20 | 2.8 | 50% | 300 | 40 | 40 | 3.4 |
| 40% | 300 | 12 | 5 | 3.3 | 40% | 300 | 20 | 20 | 3.5 | 40% | 300 | 40 | 40 | 4.0 |
| 30% | 300 | 12 | 5 | 4.1 | 30% | 300 | 20 | 20 | 4.3 | 30% | 300 | 40 | 40 | 4.6 |
| 20% | 300 | 12 | 5 | 4.9 | 20% | 300 | 20 | 20 | 5.0 | 20% | 300 | 40 | 40 | 5.2 |
| 10% | 300 | 12 | 5 | 5.7 | 10% | 300 | 20 | 20 | 5.8 | 10% | 300 | 40 | 40 | 5.9 |
| 90% | 350 | 12 | 5 | -2.0 | 90% | 350 | 20 | 20 | -1.4 | 90% | 350 | 40 | 40 | -0.4 |
| 80% | 350 | 12 | 5 | -1.1 | 80% | 350 | 20 | 20 | -0.6 | 80% | 350 | 40 | 40 | 0.4 |
| 70% | 350 | 12 | 5 | -0.1 | 70% | 350 | 20 | 20 | 0.3 | 70% | 350 | 40 | 40 | 1.1 |
| 60% | 350 | 12 | 5 | 0.8 | 60% | 350 | 20 | 20 | 1.2 | 60% | 350 | 40 | 40 | 1.9 |
| 50% | 350 | 12 | 5 | 1.8 | 50% | 350 | 20 | 20 | 2.1 | 50% | 350 | 40 | 40 | 2.7 |
| 40% | 350 | 12 | 5 | 2.7 | 40% | 350 | 20 | 20 | 3.0 | 40% | 350 | 40 | 40 | 3.4 |
| 30% | 350 | 12 | 5 | 3.7 | 30% | 350 | 20 | 20 | 3.8 | 30% | 350 | 40 | 40 | 4.2 |
| 20% | 350 | 12 | 5 | 4.6 | 20% | 350 | 20 | 20 | 4.7 | 20% | 350 | 40 | 40 | 5.0 |
| 10% | 350 | 12 | 5 | 5.5 | 10% | 350 | 20 | 20 | 5.6 | 10% | 350 | 40 | 40 | 5.7 |

**Property Methods and Property Estimation.** Aspen Plus V14 was used for process modeling. Given the non-ideality of the components used in the simulation, the Redlich-Kwong-Soave equation with the Boston-Mathias extension (RKS-BM) property method was chosen as the global calculation method.

All pure component thermodynamic and physical properties were taken from Aspen Plus databanks or estimated using the National Institute of Science and Technology ThermoDataEngine (NIST-TDE) capabilities built into the Aspen Plus software package. Binary interaction parameters were also taken from Aspen Plus databanks or estimated using UNIFAC and fit to the RKS-BM property method.

**Process Economics.** Economic assumptions were consistent with other recent TEA modeling work, including cost year basis (2020), tax rate (21%), on-stream time (90%), and plant startup time (0.5 years). For each process simulation, material, and energy flows calculated by the Aspen Plus process model were imported into an Excel spreadsheet, accounting for capital and operational costs. Given multiple products in the biomass pyrolysis facility, the minimum selling prices of biochar and bio-oil were in the range of $188-260/t for slow pyrolysis and $6.49-9.68/GGE in the case of fast pyrolysis, based on the co-products revenues as determined using a DCFROR analysis to achieve a net present value (NPV) of zero assuming an after-tax rate of return of 10% over the 30-year lifespan of the refinery.

**Capital Costs.** All feedstock handling unit operations were similar to Yadav *et al.*[14] or from vendor quotes for custom-design costs. Other capital equipment base costs, scaling exponents, and installation factors were identical to that of the Dutta *et al.*[15] report adjusted to a 2020 cost index. In the product recovery section, the costs of pumps, compressors, and flash drums were calculated from the Aspen Capital Cost Evaluator (ACCE) V14 using flowrates and operating conditions imported from the results of the Aspen Plus simulation and standard refinery operating conditions with default costing assumptions.

**Operating Costs**. Variable operating costs for raw materials, wastes, utilities, and process byproducts were determined using flow rates from the Aspen Plus process simulation.

## References


(1) Mohan, D.; Pittman, C. U. Jr.; Steele, P. H. Pyrolysis of Wood/Biomass for Bio-Oil: A Critical Review. *Energy Fuels* **2006**, *20* (3), 848–889. https://doi.org/10.1021/ef0502397.

(2) Spokas, K. A. Review of the Stability of Biochar in Soils: Predictability of O:C Molar Ratios. *Carbon Manag.* **2010**, *1* (2), 289–303. https://doi.org/10.4155/cmt.10.32.

(3) IBI. *Standardized Product Definition and Product Testing Guidelines for Biochar That Is Used in Soil*; Product Definition and Specification Standards IBI-STD-2.1. https://biochar-international.org/wp-content/uploads/2018/04/IBI_Biochar_Standards_V2.1_Final.pdf.

(4) Hale, S. E.; Lehmann, J.; Rutherford, D.; Zimmerman, A. R.; Bachmann, R. T.; Shitumbanuma, V.; O'Toole, A.; Sundqvist, K. L.; Arp, H. P. H.; Cornelissen, G. Quantifying the Total and Bioavailable Polycyclic Aromatic Hydrocarbons and Dioxins in Biochars. *Environ. Sci. Technol.* **2012**, *46* (5), 2830–2838. https://doi.org/10.1021/es203984k.

(5) Lehmann, J.; Joseph, S. *Biochar for Environmental Management: Science and Technology*.

(6) Filiberto, D. M.; Gaunt, J. L. Practicality of Biochar Additions to Enhance Soil and Crop Productivity. *Agriculture* **2013**, *3* (4), 715–725. https://doi.org/10.3390/agriculture3040715.

(7) Bridgwater, A. V. Renewable Fuels and Chemicals by Thermal Processing of Biomass. *Chem. Eng. J.* **2003**, *91* (2), 87–102. https://doi.org/10.1016/S1385-8947(02)00142-0.

(8) Bridgwater, A. V. Review of Fast Pyrolysis of Biomass and Product Upgrading. *Biomass Bioenergy* **2012**, *38*, 68–94. https://doi.org/10.1016/j.biombioe.2011.01.048.

(9) Oasmaa, A.; Czernik, S. Fuel Oil Quality of Biomass Pyrolysis OilsState of the Art for the End Users. *Energy Fuels* **1999**, *13* (4), 914–921. https://doi.org/10.1021/ef980272b.

(10) *The Right Course of Action: Climate Policies in a Shock-Prone World*. IMF. https://www.imf.org/en/News/Articles/2023/12/02/sp-md-cop28-business-philanthropy-climate-forum (accessed 2025-10-24).

(11) Fuss, S.; Lamb, W. F.; Callaghan, M. W.; Hilaire, J.; Creutzig, F.; Amann, T.; Beringer, T.; de Oliveira Garcia, W.; Hartmann, J.; Khanna, T.; Luderer, G.; Nemet, G. F.; Rogelj, J.; Smith, P.; Vicente, J. L. V.; Wilcox, J.; del Mar Zamora Dominguez, M.; Minx, J. C. Negative Emissions—Part 2: Costs, Potentials and Side Effects. *Environ. Res. Lett.* **2018**, *13* (6), 063002. https://doi.org/10.1088/1748-9326/aabf9f.

(12) *Why Improving Measurement Technology Is Key to Scaling Carbon Dioxide Removal*. Synapse. https://www.synapse.com/the-edge/why-improving-measurement-technology-is-key-to-scaling-carbon-dioxide-removal/ (accessed 2025-08-07).

(13) *Quantifying delivered carbon removal as a buyer of early technologies*. Frontier. https://frontierclimate.com/writing/quantifying-delivered-cdr (accessed 2025-08-07).

(14) Yadav, G.; Singh, A.; Dutta, A.; Uekert, T.; S. DesVeaux, J.; R. Nicholson, S.; C.D. Tan, E.; Mukarakate, C.; A. Schaidle, J.; J. Wrasman, C.; C. Carpenter, A.; M. Baldwin, R.; Román-Leshkov, Y.; T. Beckham, G. Techno-Economic Analysis and Life Cycle Assessment for Catalytic Fast Pyrolysis of Mixed Plastic Waste. *Energy Environ. Sci.* **2023**, *16* (9), 3638–3653. https://doi.org/10.1039/D3EE00749A.

(15) Dutta, A.; Talmadge, M.; Hensley, J.; Worley, M.; Dudgeon, D.; Barton, D.; Groenendijk, P.; Ferrari, D.; Stears, B.; Searcy, E. M.; Wright, C. T.; Hess, J. R. *Process Design and Economics for Conversion of Lignocellulosic Biomass to Ethanol: Thermochemical Pathway by Indirect Gasification and Mixed Alcohol Synthesis*; NREL/TP--5100-51400; National Renewable Energy Laboratory (NREL), Golden, CO (United States), 2011. https://doi.org/10.2172/1219435.
